# Developing and Validating a Context-Sensitive Scale of Excellence-Driven Behavior in Public Universities: A Mixed-Methods Psychometric Study

**DOI:** 10.3390/bs16060950

**Published:** 2026-06-09

**Authors:** Zhe Cui, Chenxi Sun, Xinan Zhao, Ningning Chen

**Affiliations:** 1 School of Business Administration, Northeastern University, Shenyang 110169, China; 2110465@stu.neu.edu.cn (Z.C.); xnzhao@mail.neu.edu.cn (X.Z.); chennn@sie.edu.cn (N.C.); 2 School of Business, Shenyang University, Shenyang 110169, China

**Keywords:** mixed-methods research, scale development, psychometric validation, measurement development, excellence-driven behavior, public universities

## Abstract

Quantitative behavioral research depends on clear construct specification and psychometrically sound measurement tools, especially when emerging constructs are examined in context-sensitive organizational settings. This study developed and validated a scale of excellence-driven behavior among faculty and staff in Chinese public universities. A mixed-methods measurement-development design was used. First, the construct domain was derived from Excellence-Driven theory and contextualized within public universities. Second, qualitative evidence from semi-structured interviews and open-ended questionnaires using the Critical Incident Technique was used to generate and refine behavioral indicators. Third, the resulting instrument was examined through item analysis, exploratory factor analysis, confirmatory factor analysis, and reliability and validity assessment. The findings supported a two-dimensional structure consisting of Excellence-Driven Cognition and Learning and Excellence-Driven Display. The scale showed acceptable evidence of internal structure, internal consistency, convergent validity, discriminant validity between the two dimensions, and preliminary criterion-related validity. These results provide initial psychometric support for the use of the scale in future research on excellence-driven behavior among faculty and staff in Chinese public universities, while further evidence regarding temporal stability, measurement invariance, and broader empirical distinction from adjacent constructs is still needed.

## 1. Introduction

Quantitative methods play a central role in advancing behavioral science research in psychology, education, and organizational studies because they enable researchers to test theories, examine behavioral patterns, and generate evidence-based conclusions. At the same time, rigorous quantitative inquiry depends on the availability of conceptually clear and psychometrically sound measurement instruments. When a construct has not yet been adequately operationalized, theoretical progress and empirical testing are both constrained. In this sense, scale development is not merely a technical procedure but a foundational step in behavioral science research, especially when scholars seek to examine context-sensitive behaviors in formal institutional settings.

Since its introduction, Excellence-Driven theory has developed mainly around conceptual, evaluative, and managerial issues (Zhao et al., 2006). Existing studies suggest that it concerns individuals’ proactive efforts to understand and enact excellence by identifying relevant standards, developing personal strengths, and translating them into superior outcomes (Liu et al., 2013; Peterson & Seligman, 2004; Zhao et al., 2012). Prior work has emphasized the conceptual logic of Excellence-Driven, the methodological framework for Excellence-Driven evaluation, and the managerial framework for Excellence-Driven practice, including systems that recognize individual strengths, respect uniqueness, and encourage employees to identify, develop, and apply their strengths at work (Huang et al., 2022; Yang et al., 2020; Zhao & Kim, 2020; Zhao et al., 2023). However, most prior work has focused on evaluation systems and management implications rather than on excellence-driven behavior as a measurable work behavior in formal organizational settings.

Public universities provide a theoretically meaningful context for examining this behavior because they are knowledge-intensive and highly structured organizations in which evaluation, promotion, and role expectations are relatively explicit (Démurger et al., 2012; Flattau et al., 2006; Guan & Blair, 2022; Planells-Aleixandre et al., 2025). In Chinese public universities, faculty and staff often work under formalized performance systems while also facing the possibility that their professional capabilities are not fully utilized (Démurger et al., 2012; Erdogan et al., 2011; Fan & Shang, 2025; Maynard et al., 2015; Wibowo et al., 2026). From the perspective of social exchange theory and conservation of resources theory, such underutilization may contribute to frustration, strain, disengagement, and burnout, thereby making positive behavioral resources especially important in this setting (Bakker et al., 2003; Bochoridou & Gkorezis, 2023; Chambel et al., 2021; Cheng et al., 2020, 2021; Cropanzano & Mitchell, 2005; Erdogan & Bauer, 2021; Harari et al., 2017; Hobfoll, 2001; Khan et al., 2024; Li et al., 2019; Lin et al., 2022; Ma et al., 2019; Maslach et al., 2001; Maynard & Parfyonova, 2013; Robbins et al., 2012; Shang et al., 2024; Weng et al., 2018; Zhang et al., 2024). In such a setting, a construct capturing whether organizational members proactively clarify standards, develop relevant capabilities, and translate them into visible contributions is especially relevant.

Despite the relevance of this context, three gaps remain. First, excellence-driven behavior has rarely been operationalized as a distinct behavioral construct in formal organizational settings. Second, the only existing scale was developed in a virtual community context, which limits its applicability to public universities (Hou et al., 2018). Third, no validated context-sensitive measure currently exists for faculty and staff in Chinese public universities. These gaps limit both theoretical development and empirical testing and make it difficult to examine excellence-driven behavior as a potentially meaningful positive work behavior in institutional settings. The conceptual distinction between excellence-driven behavior and adjacent constructs is discussed in Section 2.4.

**Figure 1:**
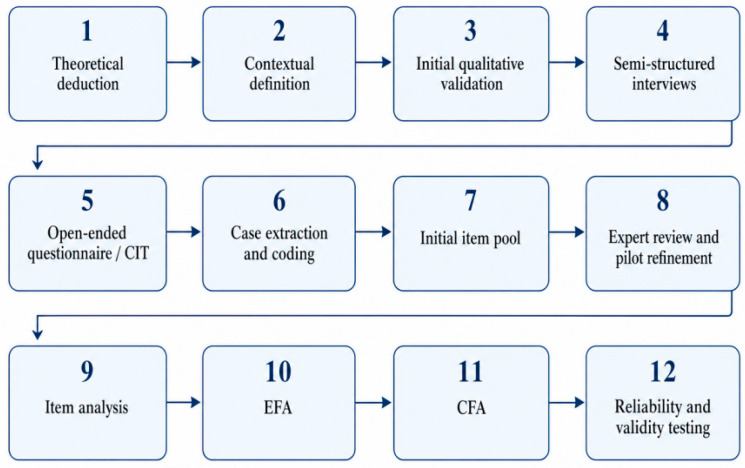
Mixed-Methods Scale Development Process for Excellence-Driven Behavior.

Accordingly, the present study uses a mixed-methods design to define excellence-driven behavior in the context of Chinese public universities, identify its dimensional structure, and validate a scale for its assessment. Specifically, the study pursues three objectives: first, to define excellence-driven behavior in the public university context; second, to identify its dimensional structure through the integration of deductive reasoning and qualitative evidence; and third, to validate the resulting instrument using item analysis, exploratory factor analysis, confirmatory factor analysis, and reliability and validity assessment. This design follows the broader methodological argument that emerging constructs requiring contextual adaptation are best developed through the integration of qualitative exploration and quantitative psychometric validation (Boateng et al., 2018; Glaser et al., 1968; Hinkin, 1995, 2005; Koller et al., 2017; Ou et al., 2014). By integrating qualitative construct development with quantitative validation, this study contributes not only a new behavioral assessment tool but also a methodological example of how emerging constructs can be operationalized for behavioral science research. To provide an overview of the research design, Figure 1 illustrates the mixed-methods scale development process for excellence-driven behavior adopted in the present study.

## 2. Theoretical Background and Construct Definition

### 2.1. The Concept of Excellence-Driven Behavior

The concept of excellence-driven behavior originates in Excellence-Driven theory and refers to a positive behavioral pattern through which individuals proactively explore, understand, and apply standards or principles that guide effective action, while demonstrating excellence in practice (Peterson & Seligman, 2004; Zhao et al., 2006, 2012). Unlike conventional competitive behavior, which emphasizes advantage in resource acquisition, excellence-driven behavior highlights constructive alignment between individual strengths and contextually valued standards.

From this perspective, excellence-driven behavior is not simply a matter of personal ambition or generalized self-improvement. Rather, it reflects a process in which individuals actively identify what counts as excellence in a given context, develop the capabilities required to achieve it, and enact these capabilities in ways that are visible and socially meaningful within the organization. This understanding makes the construct especially relevant for behavioral research in structured institutional settings.

### 2.2. The Connotation of Excellence-Driven Behavior

In the present study, the connotation of excellence-driven behavior is understood through three closely connected elements: clarifying relevant standards, developing or mobilizing capabilities, and enacting excellence in visible and socially recognized ways (Liu et al., 2013; Zhao et al., 2012). In other words, excellence-driven behavior is not confined to inward motivation or ability acquisition alone. Its behavioral meaning lies in the process through which individuals actively align themselves with organizationally valued standards and translate that alignment into observable contributions.

This process-oriented understanding is especially important because excellence is not a purely personal judgment. In organizational contexts, standards of excellence are shaped by institutional expectations, evaluation systems, role requirements, and promotion structures. Accordingly, the specific content of excellence-driven behavior must be interpreted in relation to the norms and value orientations of the organization in which it occurs.

### 2.3. Existing Research and Measurement Approaches to Excellence-Driven Behavior

Empirical research on excellence-driven behavior remains limited. Liu et al. (2013) examined its core characteristics through the evolutionary path of “knowing” and “doing.” Their analysis suggested that excellence-driven behavior helps individuals develop distinctive strengths while also emphasizing harmony among individuals, organizations, and environments, thereby supporting sustainable development. Chen et al. (2022), using grounded theory, explored the antecedents and mechanisms of excellence-driven behavior among exploratory and research-oriented employees. Their findings showed that environmental fit, individual cognition, and supportive contextual factors are important antecedents, and that cognitive factors directly drive the emergence and reinforcement of excellence-driven behavior.

With regard to measurement, Hou et al. (2018) appears to be the only study to have explicitly examined the dimensional structure and formation mechanism of excellence-driven behavior. Focusing on corporate virtual communities, they developed a two-dimensional, nine-item scale consisting of exploratory behavior (six items) and superior performance display behavior (three items). However, the applicability of this scale is constrained by its specific research context. Several items are closely tied to platform-based activities such as “liking,” “posting,” “sharing,” and “interacting,” which limits the instrument’s suitability for formal organizational settings and weakens its contextual generalizability.

### 2.4. Research Gaps in Excellence-Driven Behavior and the Public University Context

Existing studies on excellence-driven behavior remain limited in three respects. First, the construct has rarely been examined as a distinct behavioral pattern in formal organizational settings. Second, the only existing measurement instrument was developed in a virtual community context, which limits its applicability to knowledge-intensive and institutionally structured organizations. Third, no validated context-sensitive measure currently exists for faculty and staff in Chinese public universities, where evaluation standards, promotion requirements, and role expectations are relatively explicit.

Public universities constitute a particularly important context for addressing these gaps. Unlike many other organizations, public universities are knowledge-intensive, mission-oriented institutions that simultaneously pursue educational quality, research productivity, public service, and long-term social value (Démurger et al., 2012; Flattau et al., 2006; Guan & Blair, 2022; Planells-Aleixandre et al., 2025). Faculty and staff in such settings are often highly educated and professionally capable, yet they also work within relatively standardized role structures, bureaucratic procedures, and formalized evaluation systems (Cao et al., 2025; Ruan et al., 2024; Wen & Marginson, 2023). Under these conditions, individual strengths and developmental initiative may not always be fully reflected by routine job requirements alone. For this reason, excellence-driven behavior is especially meaningful in public universities because it concerns whether organizational members can proactively clarify standards, develop relevant capabilities, and convert them into visible and value-consistent contributions.

At the same time, excellence-driven behavior should not be treated as a simple synonym for other positive work behaviors. Although it is related to constructs such as proactive behavior, organizational citizenship behavior, and learning behavior, its conceptual emphasis lies in the combined process of clarifying organization-specific standards, developing relevant capabilities, and translating them into visible and value-consistent contributions. Compared with proactive behavior, it places stronger emphasis on alignment with formal evaluation and advancement criteria (Baer & Frese, 2003; Griffin et al., 2007). Compared with organizational citizenship behavior, its core is not discretionary helping per se, even though helping may appear as one behavioral expression (Organ, 1988; Podsakoff et al., 2000). Compared with learning behavior, learning is not an end in itself but part of a broader process through which acquired knowledge and skills are converted into recognized contribution (Peng et al., 2022). These gaps collectively support the need for a context-sensitive measurement-development study.

If excellence-driven behavior is to be examined rigorously in public universities, it must first be clearly defined at the behavioral level and translated into an instrument that reflects the institutional realities of this setting. The present study addresses this need by integrating conceptual deduction, qualitative evidence, and quantitative psychometric validation in order to develop a context-sensitive scale of excellence-driven behavior among faculty and staff in Chinese public universities. A concise conceptual comparison between excellence-driven behavior and adjacent constructs is provided in Supplementary Table S18.

## 3. Materials and Methods

This study used a multi-stage mixed-methods measurement-development design to conceptualize and validate excellence-driven behavior among faculty and staff in public universities. The design combined theoretical deduction, qualitative item generation, expert review, pilot refinement, and quantitative psychometric validation. First, the construct domain was specified based on Excellence-Driven theory and the organizational characteristics of Chinese public universities. Second, qualitative data from semi-structured interviews and open-ended questionnaires incorporating the Critical Incident Technique were used to generate and classify behavioral indicators. Third, the initial item pool was reviewed by experts and refined through a pilot test. Finally, the resulting scale was evaluated using item analysis, exploratory factor analysis, confirmatory factor analysis, and reliability and validity assessment.

The study was conducted in accordance with the Declaration of Helsinki and was approved by the Ethics Committee of Northeastern University (No. NEU-BA-202312-018; 19 December 2023). Participation was voluntary and anonymous. Participants were informed that the data would be used only for research purposes and that they could withdraw from the study at any time. Informed consent was obtained from all participants.

Following psychometric scale development procedures recommended by Hinkin (2005), the study was carried out in three phases. In the first phase, a literature review and logical deduction were used to generate a preliminary definition of excellence-driven behavior in the context of public universities. In the second phase, original qualitative data were collected through interviews and open-ended questionnaires and then organized into an initial pool of items. In the third phase, the preliminary questionnaire was tested and further refined through qualitative and quantitative procedures, resulting in the final scale.

### 3.1. Construct Specification and Contextualization

Excellence-driven behavior is a relatively abstract behavioral construct. For this reason, it is necessary to situate it in a concrete organizational setting before proceeding to item generation and psychometric validation. In the present study, construct specification served two purposes: first, to clarify how the general idea of excellence-driven behavior should be interpreted in public universities; and second, to establish a context-sensitive conceptual basis for the subsequent qualitative phase.

#### 3.1.1. Characteristics of Public Universities

Public universities in China are characterized by relatively stable organizational structures, formalized procedures, and clearly structured systems of evaluation and promotion (Cao et al., 2025; Démurger et al., 2012; Ruan et al., 2024). Administrative ranks and professional titles are usually classified in a clear and institutionalized manner, and advancement typically follows explicit standards and procedures. At the same time, members in these organizations often work within long-term employment arrangements, with comparatively strong job security and relatively low turnover (Wen & Marginson, 2023).

These institutional features make public universities especially suitable for specifying excellence-driven behavior. In such settings, organizational members are not only expected to fulfill routine work requirements but also to understand formal standards, develop relevant capabilities, and translate these into visible contributions that are recognized within the institution. Because standards of achievement, evaluation, and advancement are relatively explicit, public universities provide a context in which the behavioral meaning of “excellence” can be identified more clearly than in loosely structured environments.

#### 3.1.2. Phases of Excellence-Driven Behavior

In the present study, excellence-driven behavior is conceptualized as a connected process rather than as a single isolated act. At a general level, this process can be understood in two closely related phases. The first phase involves clarifying and internalizing the standards, expectations, and pathways through which excellence is defined in a given organizational setting. The second phase involves applying acquired knowledge, skills, and understanding in order to demonstrate excellence in practice.

These two phases are analytically distinct but empirically connected. The first phase concerns orientation: individuals seek to understand what counts as excellence and what capabilities are needed to achieve it. The second phase concerns enactment: individuals translate this orientation into visible and organizationally meaningful behavior. In this sense, excellence-driven behavior is neither purely cognitive nor purely performative. Rather, it links standards clarification, capability development, and behavioral enactment.

#### 3.1.3. The Two Phases of Excellence-Driven Behavior in Public Universities

In public universities, the first phase of this process is reflected in members’ efforts to understand evaluation criteria, promotion requirements, and the knowledge and skills needed for competent and high-quality work. Such understanding may be developed through institutional documents, internal communication channels, formal mentoring, observation of experienced colleagues, and participation in internal or external training. Because university roles are often clearly differentiated and evaluation systems are relatively visible, members can actively identify the standards and pathways that structure advancement and recognition in the organization.

The second phase is reflected in how members use what they have learned to improve performance, attain recognition, support colleagues, and contribute to institutional goals. In public universities, excellence is not displayed only through individual achievement in a narrow sense. It may also be enacted through visible competence, strong appraisal outcomes, advancement in rank or title, the sharing of useful knowledge and experience, and constructive contributions to others. Thus, the organizational setting makes it possible to observe how standards clarification and capability development are translated into contextually valued forms of behavioral display.

#### 3.1.4. Definition of Excellence-Driven Behavior Among Faculty and Staff in Public Universities

Based on the above contextualization, excellence-driven behavior among faculty and staff in public universities is defined in the present study as a positive work behavior through which organizational members proactively clarify standards and expectations relevant to excellence, develop or mobilize the knowledge and skills needed for high-quality work and professional advancement, and translate these resources into visible, value-consistent contributions within the organization.

This working definition was used as the conceptual basis for the qualitative phase of the study. It guided the development of the interview protocol and the open-ended questionnaire prompts, and it provided the interpretive framework through which raw behavioral materials were screened, coded, and categorized. In this way, the construct specification stage did not function as a purely abstract theoretical exercise, but as a bridge between conceptual definition and empirical item generation.

To examine whether this concept was consistent with actual organizational practice, the study conducted an initial round of qualitative validation before formal scale development. Seventeen faculty and staff members from four public universities in Liaoning Province, China, participated in these interviews. The sample included seven men and ten women; nine administrative staff members, six teachers, and two technical workers; two participants under 25 years old, nine aged 26–35, four aged 36–45, and two aged 46 or above; four participants with doctoral degrees, eleven with master’s degrees, and two with bachelor’s degrees. In terms of years of service, four participants had worked for 1–3 years, eight for 4–6 years, three for 7–9 years, and two for 10 years or more.

At the beginning of each interview, participants were introduced to the basic concept of excellence-driven behavior and reminded of the voluntary, anonymous, and research-only nature of the study. They were then asked to recall specific instances in which they or their colleagues had displayed behaviors consistent with excellence-driven behavior in the organization. When such cases were reported, participants were invited to describe the behavioral process and specific content in detail.

After the interviews, the audio recordings were transcribed and analyzed as textual data. A total of 57 behavioral examples were initially extracted. Cases that did not conform to the conceptual definition of excellence-driven behavior, such as those involving competition or arrogance, were removed, leaving 53 valid examples. Similar or identical examples were then merged, resulting in 25 representative behavioral cases. Through further classification and summarization, several recurring themes were identified, including actively understanding promotion pathways, clarifying promotion requirements, tracking organizational development priorities, preparing materials needed for promotion, and striving for advancement in administrative rank or professional title. These findings provided preliminary support for the applicability and contextual relevance of the construct in public universities.

### 3.2. Qualitative Item Generation

On the basis of the working definition established through construct specification and contextualization, this study employed semi-structured interviews and open-ended questionnaires to collect qualitative evidence from faculty and staff in public universities. All qualitative materials in the present study were collected and analyzed in Chinese, which was the working language of the participants and the language used in the actual study administration. Accordingly, the initial item pool and subsequent scale refinement were all based on Chinese-language materials. The purpose of this phase was to identify behaviorally meaningful expressions of excellence-driven behavior in this setting, generate an initial item pool, and develop a measurement structure grounded in both conceptual reasoning and empirical materials.

It is important to distinguish between the process logic of the construct and the dimensional structure of the scale. The process logic refers to the broader sequence through which organizational members clarify standards, develop relevant capabilities, and translate them into visible contribution. By contrast, the dimensional structure refers to how this process was operationalized for measurement purposes. In the present study, the qualitative analysis ultimately supported two dimensions: Excellence-Driven Cognition and Learning, which captures the standards-oriented and capability-development component of the construct, and Excellence-Driven Display, which captures the visible enactment of excellence in organizational practice.

#### 3.2.1. In-Depth Interviews

Prior to conducting the formal interviews, an interview guide was developed. Based on the conceptual definition of excellence-driven behavior among faculty and staff in public universities and the two-phase process identified through logical deduction, a semi-structured interview guide containing 11 primary questions was prepared. Example questions included: “What are your career plans and goals?”, “Through what channels or methods do you pursue these career goals?”, and “How do you demonstrate your excellence within the organization?” Additional probing questions were asked according to participants’ responses in order to elicit detailed behavioral descriptions and concrete examples.

Before the formal interviews were conducted, the interview guide was pilot-tested with 6 faculty and staff members from two public universities in Liaoning Province, China, including 2 administrative staff members, 3 teachers, and 1 technical worker. The purpose of this pilot phase was to assess the clarity, comprehensibility, and contextual appropriateness of the interview questions. Based on participants’ feedback, 3 questions were reworded for greater clarity, 1 follow-up question was added, and the order of 2 questions was adjusted to improve the flow of the interview. The pilot participants were not included in the final formal interview sample.

The formal interviews were then conducted with 47 faculty and staff members from five public universities in Liaoning Province, China. Among them, 17 were male and 30 were female. Participants ranged in age primarily from 23 to 46 years, with an average age of 36.9 years. In terms of educational background, 5 held bachelor’s degrees, 25 held master’s degrees, and 17 held doctoral degrees. In terms of job type, the interview sample consisted of 22 administrative staff members, 20 full-time teachers, and 5 technical workers. Each interview lasted between 40 and 60 min, with an average duration of approximately 51 min. All interviews were audio-recorded, transcribed verbatim, and supplemented with field notes. Following the principle of theoretical sampling, unclear or incomplete content was subsequently checked with participants to ensure the accuracy and completeness of the behavioral data. Finally, four members of the research team independently reviewed and analyzed the interview materials to extract concrete examples of excellence-driven behavior among members of public universities.

#### 3.2.2. Open-Ended Questionnaire

To further enhance theoretical saturation and assess whether additional data would yield substantively new categories, the Critical Incident Technique (CIT) was incorporated into an open-ended questionnaire. Several key questions from the interview guide were adapted into written prompts, including questions such as: “What are your career plans and goals?”, “How do you identify the requirements needed to achieve these goals?”, “What actions have you taken to pursue these goals?”, “How do you improve your work-related knowledge and skills?”, “How do you usually deal with difficulties at work?”, “How do you demonstrate the knowledge and skills you have acquired?”, and “How do you display excellence-driven behavior within the organization?” Respondents were asked to describe critical incidents and list relevant keywords.

CIT is widely used in scale development to identify conceptual prototypes from concrete behavioral evidence (Flanagan, 1954). Because it relies on respondents’ descriptions of actual events and involves systematic coding and categorization, it is particularly useful for inductively refining constructs whose dimensions may not be fully captured through logical deduction alone (Bitner et al., 1990). As excellence-driven behavior is a process-oriented organizational behavior that can be expressed through concrete work events, the inclusion of CIT helped capture its behavioral manifestations more comprehensively in the public university context.

Paper-based questionnaires and online questionnaires distributed through Questionnaire Star, a professional Chinese survey platform, were administered to 50 faculty and staff members in public universities in Liaoning Province, China, yielding 41 valid responses. The respondents included 18 men and 23 women; 22 administrative staff members, 16 teachers, and three technical workers; 10 participants under 25 years old, 22 aged 26–35, seven aged 36–45, and two aged 46 years or above. In terms of education, nine respondents held doctoral degrees, 28 held master’s degrees, and four held bachelor’s degrees. With regard to years of service, 11 had worked for 1–3 years, 23 for 4–6 years, five for 7–9 years, and two for 10 years or more.

Theoretical saturation was assessed across the interview and open-ended questionnaire materials rather than within a single source alone. During the later stage of data collection and coding, newly collected interview narratives no longer generated substantively new labels or higher-order categories, and the additional open-ended questionnaire responses primarily repeated patterns that had already emerged from the interview data. The coding framework was refined iteratively during analysis and remained stable in the final stage of coding. On this basis, the research team judged that theoretical saturation had been reached when continued data collection produced repetition and elaboration rather than substantively new conceptual content.

#### 3.2.3. Case Extraction and Classification

The in-depth interviews and open-ended questionnaires yielded a total of 231 valid original statements. These statements were systematically screened, summarized, and classified in order to develop a structural model of excellence-driven behavior and to identify representative behavioral cases.

To enhance the rigor of the qualitative coding process, a subset of the original statements was independently coded prior to full-scale classification. Specifically, 20% of the statements (*n* = 46) were randomly selected and independently coded by two members of the research team. The initial coding agreement rate was 95.65%, and Cohen’s kappa was 0.94, indicating satisfactory inter-coder reliability in case extraction, label assignment, and category classification. Discrepancies were subsequently discussed and resolved through consultation with the remaining members of the research team, after which the refined coding framework was applied to the full dataset.

First, concrete behavioral cases were extracted from the original statements. Statements that were clearly inconsistent with the connotation of excellence-driven behavior, such as eccentric behavior, malicious competition, or blind overconfidence, were removed. This resulted in an initial conceptual pool of 63 concrete behavioral cases. Representative examples included: “I review documents related to professional title requirements,” “I monitor recent organizational developments,” “I think about how to complete my tasks with high quality,” “I strive for promotion in professional title,” “I study the knowledge required for my position,” and “I willingly help colleagues within my capacity.”

Second, the 63 cases were merged when they described identical or highly similar events, producing 37 cases. Third, each case was coded according to its content, resulting in 21 labels. Fourth, labels with similar meanings were grouped into broader categories, yielding eight categories. Finally, based on the results of logical deduction and the two-phase process of excellence-driven behavior, these eight categories were organized into two dimensions: Excellence-Driven Cognition and Learning, and Excellence-Driven Display. In this framework, learning encompasses both exploratory learning processes and the mastery of relevant knowledge and skills.

To enhance the transparency of category development, the coding process proceeded from concrete behavioral expression to increasingly abstract conceptual grouping. First, statements that clearly contradicted the connotation of excellence-driven behavior were excluded. Second, behaviorally similar statements were merged to reduce redundancy while preserving conceptual coverage. Third, the remaining cases were assigned descriptive labels based on their substantive content. Fourth, these labels were grouped into broader categories by comparing similarities in behavioral meaning, functional role, and relevance to the theoretically derived process of excellence-driven behavior. Finally, the resulting categories were mapped onto the emerging dimensional structure. This procedure made it possible to move from raw behavioral evidence to conceptually coherent measurement dimensions while maintaining consistency with the logic derived from Excellence-Driven theory.

More specifically, the final two dimensions did not emerge as arbitrary statistical groupings. Rather, they reflected the convergence between deductive reasoning and inductive evidence. Excellence-Driven Cognition and Learning captures how organizational members clarify standards, seek information, invest in capability development, and master relevant knowledge and skills. Excellence-Driven Display captures how these internalized standards and acquired capabilities are enacted through visible performance, advancement, sharing, and contribution to others. Together, the two dimensions represent a context-sensitive operationalization of excellence-driven behavior among faculty and staff in public universities.

Table 1 presents the hierarchical relationships among labels, categories, and dimensions.

### 3.3. Expert Review, Pilot Refinement, and Initial Scale Formation

The 21 labels identified through qualitative coding were then transformed into draft questionnaire items for expert review and initial scale formation. The originator of Excellence-Driven theory, members of the research team, and selected faculty and staff members familiar with the concept were invited to review the initial items in terms of redundancy, clarity, and comprehensibility. The expert review was conducted in 2 rounds and involved 9 reviewers, including 3 scholars specializing in Excellence-Driven theory, 4 researchers in organizational behavior and psychometrics, and 2 senior faculty members and administrators from public universities. In the first round, the reviewers evaluated the draft items with regard to conceptual representativeness, redundancy, wording clarity, and contextual appropriateness. In the second round, the revised items were re-examined to confirm content coverage, eliminate residual ambiguity, and ensure alignment with the emerging dimensional structure of excellence-driven behavior.

To provide more transparent evidence of content validity, the nine expert reviewers evaluated the preliminary 21-item pool in terms of relevance, clarity, and representativeness using separate four-point rating scales. Item-level content validity indices (I-CVI) were calculated as the proportion of experts assigning a rating of 3 or 4 to a given item. For the preliminary 21-item pool, the I-CVI values ranged from 0.67 to 1.00 for relevance, from 0.78 to 1.00 for clarity, and from 0.67 to 1.00 for representativeness, while the corresponding S-CVI/Ave values were 0.92, 0.90, and 0.87, respectively. Table 2 summarizes the content validity assessment and qualitative refinement process that led from the preliminary 21-item pool to the final 11-item scale, while the full item-level results remain available in Supplementary Table S1.

The transition from qualitative categories to questionnaire items followed three principles: conceptual representativeness, linguistic clarity, and contextual relevance. Based on the combined quantitative ratings and qualitative feedback from the two rounds of expert review, four items were merged because of conceptual overlap, two items were deleted because they were judged to be comparatively narrow or less representative of the target construct, and five items were reworded to improve clarity and contextual appropriateness. This process resulted in a 15-item test version, including nine items for Excellence-Driven Cognition and Learning and six items for Excellence-Driven Display.

The resulting 15-item questionnaire was then pilot-tested with eight faculty and staff members from two public universities who had not participated in the formal interviews. The pilot test served as a qualitative refinement step rather than as a formal psychometric test. Respondents were invited to comment on item clarity, interpretability, and contextual appropriateness. Based on their feedback, two additional items were deleted because respondents considered them difficult to interpret, four items were merged into two because of similar content, and three items were further revised to improve wording precision and contextual fit. This process resulted in the final 11-item scale. The full pilot refinement details remain available in Supplementary Table S2, and the full wording of the preliminary 15-item test scale is provided in Supplementary Materials Section S2.

Following this pilot refinement process, the wording of several retained items was further harmonized to better fit the language used by faculty and staff in public universities. In this way, the final 11-item questionnaire retained conceptual breadth while improving readability and response accuracy.

Following the final review by the proponents of Excellence-Driven theory, their team members, and relevant researchers, the formal initial measurement questionnaire was reduced to 11 items across two dimensions: 6 items for Excellence-Driven Cognition and Learning and 5 items for Excellence-Driven Display. The questionnaire used in the actual study was administered in Chinese. The English item wording reported in this manuscript was prepared for international publication and represents an English translation of the original Chinese items, rather than an independently administered English-language survey version. Accordingly, all psychometric analyses reported in this study were based on the Chinese version of the scale. To support linguistic consistency in reporting, the English wording was translated from the Chinese source items by bilingual members of the research team and checked for conceptual correspondence with the original wording. In line with Hinkin’s (2005) recommendation that each scale dimension should ideally contain four to six items, the number of items retained in the present scale was considered appropriate. No reverse-coded items were included in the final scale. All retained items were positively worded in order to maintain conceptual clarity and reduce potential confusion during item interpretation.

Excellence-Driven Cognition and Learning refers to individuals’ clear understanding of the organization’s mission, advocated values, and performance requirements, as well as their active efforts to identify, learn, and master the knowledge and skills needed to achieve excellence. This dimension constitutes the foundation of excellence-driven behavior because it defines the direction of excellence and equips individuals with the ability to enact it. The six items were as follows: “I proactively seek to understand the performance assessment and year-end evaluation standards and requirements of my position”; “I proactively seek to understand the promotion requirements and relevant policies associated with my position”; “I actively explore ways to improve the quality of my work”; “I am willing to invest time and effort in acquiring the knowledge and skills needed to improve my work performance”; “I effectively apply the knowledge and skills I have acquired to my work tasks”; and “I am eager to learn from exemplary colleagues or leaders in the organization, take their suggestions seriously, and draw on their experience.”

Excellence-Driven Display refers to the use of acquired knowledge, skills, and practical understanding to demonstrate one’s excellence in observable ways. After acquiring relevant knowledge and skills through observation, learning, and experience, individuals externalize excellence through superior performance, peer recognition, and constructive contributions to others. The five items were as follows: “I receive praise and recognition from colleagues and leaders because of my work ability”; “I can use the knowledge and skills I have mastered to achieve superior performance appraisal results”; “I can leverage my knowledge and skills to attain promotion in position or professional title”; “I often share the knowledge, experience, and methods I have acquired with my colleagues”; and “I regularly use the knowledge and skills I have acquired to help colleagues complete relevant tasks.”

The findings from the in-depth interviews and pilot refinement further suggested that the second phase of excellence-driven behavior, namely applying relevant patterns to demonstrate excellence, extended beyond individual task performance alone. It also included helping colleagues complete their work by drawing on one’s knowledge, skills, and experience. Such actions not only enhance team efficiency but also generate recognition and appreciation from both leaders and peers.

Taken together, these procedures produced a context-sensitive initial instrument that was theoretically grounded, qualitatively refined, and ready for quantitative psychometric validation. The full wording of the final 11 items is presented in Table 3.

## 4. Results

### 4.1. Data Analysis Strategy

Data analysis was conducted using SPSS 24 and AMOS 24. Item discrimination was examined using the extreme-group method and item-total correlations. The total sample was randomly divided into two equal subsamples for exploratory and confirmatory factor analyses. Sampling adequacy was assessed using the Kaiser-Meyer-Olkin index and Bartlett’s test of sphericity. Exploratory factor analysis was conducted to examine the latent structure of the scale. Factor-retention decisions were based on multiple criteria, including eigenvalues, scree plot inspection, and parallel analysis, rather than relying on the Kaiser criterion alone. Because the two latent dimensions were theoretically expected to be correlated, an oblique rotation method was adopted as the primary rotation strategy, and the direct oblimin solution was reported as the main EFA result. For robustness, the factor structure was also examined using varimax and Promax rotations, both of which yielded substantively consistent two-factor solutions. The scree plots, parallel analysis results, and alternative rotation solutions are reported in Supplementary Figures S1 and S2, and Supplementary Materials Section S3.

Before conducting factor analysis, item-level distributional properties were examined using descriptive statistics. As shown in Supplementary Tables S3 and S4, the item distributions were generally acceptable for psychometric analysis: most absolute skewness values were below 1, all absolute skewness values were below 2, and all absolute kurtosis values were below 3. Multivariate normality was further assessed for the AMOS-based analyses, and potential multivariate outliers were screened using Mahalanobis distance. Cases with p1 values below 0.001 were treated as multivariate outliers and removed prior to model estimation. Detailed results are reported in Supplementary Tables S5 and S6. Reliability and validity were assessed using Cronbach’s alpha, split-half reliability, the Spearman–Brown coefficient, composite reliability, average variance extracted, the Fornell–Larcker criterion, and the heterotrait–monotrait ratio (HTMT). To further strengthen construct validation, criterion-related validity was additionally examined using a theoretically related external construct, namely proactive behavior, through correlation and regression analyses.

The resulting instrument was then subjected to quantitative psychometric testing in order to evaluate its item quality, factor structure, reliability, and construct validity.

### 4.2. Participants and Procedure for Quantitative Validation

To further validate the preliminary questionnaire and reduce the risks of common method bias and poor response quality in online surveys, the questionnaire was administered in both paper-based and online formats through Questionnaire Star, a professional online survey platform in China. Data were collected over a 14-day period. The questionnaires were distributed to faculty and staff from seven public universities in Liaoning Province, China. Snowball sampling was used, and respondents were encouraged to forward the questionnaire to eligible colleagues. These procedural remedies were intended to reduce the likelihood of common method bias, but they cannot completely eliminate method-related inflation in self-reported data. The findings should therefore be interpreted with appropriate caution.

Participants who completed the paper-based questionnaire received a small gift, whereas those who completed the online questionnaire received a small monetary incentive. All items were measured on a five-point Likert scale ranging from 1 (strongly disagree) to 5 (strongly agree). No reverse-coded items were used in the questionnaire; all items were scored in the same positive direction. The questionnaire administered in this phase was the Chinese version of the scale. Questionnaires with substantial missing responses or incomplete scale data were treated as invalid during data screening and were excluded prior to analysis. As a result, the final analytic dataset used for psychometric testing consisted of complete cases, and no additional missing-data imputation procedure was applied. Because the survey was administered in both paper-based and online formats, we also compared respondents across administration modes. Independent-samples *t* tests showed no significant item-level differences between paper-based and online respondents across the 11 items, supporting the pooling of the two response modes for the main analyses (see Supplementary Table S17).

A total of 512 questionnaires were collected. After excluding invalid responses, such as straight-lining, clearly patterned responding, and incomplete questionnaires, 424 valid questionnaires were retained for analysis. The sample showed variation across gender, age, educational level, and years of service, providing a relatively broad basis for subsequent analyses. Detailed demographic characteristics of the full sample and the EFA and CFA subsamples are presented in Table 4.

Several procedural remedies were adopted to reduce the potential influence of common method bias. First, data were collected through both paper-based and online formats in order to diversify response channels and reduce format-specific bias. Second, respondents were informed that participation was anonymous and voluntary and that there were no right or wrong answers, which helped reduce evaluation apprehension and socially desirable responding. Finally, item wording was kept as clear and behaviorally specific as possible during scale refinement in order to minimize ambiguity and respondents’ tendency to infer expected answers. These design features helped reduce, although not eliminate, the risk of common method variance.

In addition to the main validation sample, a supplementary sample was collected to examine criterion-related validity using proactive behavior as an external construct. This supplementary sample included 105 faculty and staff members from public universities. A concise three-item proactive behavior measure was used in this phase in order to capture a theoretically related external criterion while keeping respondent burden low. The selected items were adapted from the individual task proactivity dimension proposed by Griffin et al. (2007), which conceptualizes proactive behavior as self-initiated, improvement-oriented task behavior. The demographic characteristics of this supplementary sample are reported in Supplementary Table S14.

### 4.3. Item Discrimination and Item Analysis

Item discrimination analysis was conducted to examine the discriminative power and overall item quality of the 11 items. First, the total score of the scale was calculated for each respondent, and all respondents were ranked according to their overall scores. Following the conventional upper–lower 27% extreme-group method, the top 27% of respondents were assigned to the high-score group and the bottom 27% to the low-score group. Independent-samples tests were then conducted to compare item means between the two groups. Items showing significant between-group differences were retained because they demonstrated adequate discrimination (Babbie, 2004).

The analysis was performed using SPSS 24. As shown in Table 5, all items significantly distinguished between the high-score and low-score groups. In addition, all item-total correlations were significant at the 0.01 level and ranged from 0.83 to 0.95, exceeding the commonly accepted threshold of 0.50. These results indicate that all 11 items had satisfactory discriminative ability and were appropriately retained for further analysis. For ease of interpretation, Table 5 reports both the item codes and shortened item wording, while the full wording of the final 11 items is provided in Table 3.

### 4.4. Exploratory Factor Analysis

To cross-validate the factor structure, the 424 valid questionnaires were randomly divided into two equal subsamples. One subsample (*n* = 212) was used for exploratory factor analysis (EFA), and the other subsample (*n* = 212) was used for confirmatory factor analysis (CFA). Before conducting factor analysis, the demographic characteristics of the two analytic subsamples were examined separately (see Table 4). Group comparisons confirmed that there were no significant differences between the EFA and CFA subsamples in terms of gender, age, years of service, or educational level, supporting their comparability for cross-validation purposes.

EFA was performed using SPSS 24. Bartlett’s test of sphericity was significant (χ^2^ = 2527.39, *p* < 0.001), indicating that the items shared sufficient common variance for factor analysis. The Kaiser-Meyer-Olkin (KMO) measure was 0.92, suggesting excellent sampling adequacy. In line with common scale development practice, items were retained when their primary pattern loading was at least 0.50, cross-loadings on the non-target factor were below 0.30, and the difference between the primary and secondary loading was at least 0.20. In addition, extraction communalities of 0.40 or above were treated as acceptable, and items with communalities below 0.40 would have been considered for removal (Hinkin, 2005; Lederer & Sethi, 1991). As shown in Supplementary Table S13, all retained items exceeded this threshold, with extraction communalities ranging from 0.669 to 0.913. No additional items were removed during EFA because all 11 items entering this phase had already undergone expert review and pilot refinement, and all of them met the prespecified loading, communality, and cross-loading criteria in the initial extraction. These criteria were used to identify potentially problematic items during EFA. In addition to the Kaiser criterion, scree plot inspection and parallel analysis were used to strengthen the factor-retention decision. Both the SPSS scree plot and the R-based scree plot showed a clear break after the second factor, supporting a two-factor solution. The parallel analysis likewise indicated that the number of factors to retain was two. Because the two dimensions were conceptually related, and because the oblique solutions later showed a substantial inter-factor correlation, oblique rotation was considered theoretically more appropriate than orthogonal rotation. Therefore, the direct oblimin solution was adopted as the primary EFA result, whereas the varimax and Promax solutions were examined as robustness checks. All three approaches supported the same substantive two-factor structure.

As shown in Table 6, the direct oblimin solution yielded a clear two-factor pattern matrix consistent with the theoretically derived structure of the scale. Items Item 1 to Item 6 loaded primarily on Excellence-Driven Cognition and Learning, whereas items Item 7 to Item 11 loaded primarily on Excellence-Driven Display. Primary loadings ranged from 0.77 to 1.00 for the first factor and from 0.76 to 0.95 for the second factor, while non-target loadings remained small in magnitude. The correlation between the two factors was 0.61, further supporting the appropriateness of an oblique rotation method. The substantively equivalent varimax and Promax solutions are reported in Supplementary Tables S7 and S8. To improve readability, Table 6 presents the pattern coefficients together with shortened item wording. The full wording of the final scale items is reported in Table 3. All retained items met these prespecified loading and cross-loading criteria, and no item showed a problematic loading pattern under the direct oblimin solution. Thus, the final 11-item solution reported in Table 6 was retained without further item deletion during EFA. Taken together, the eigenvalues, scree plot inspection, parallel analysis, and the substantively consistent varimax, Promax, and direct oblimin solutions converged in supporting a two-factor structure.

### 4.5. Confirmatory Factor Analysis

Confirmatory factor analysis was conducted on the second subsample, which initially included 212 cases. Prior to model estimation, multivariate normality was assessed and potential multivariate outliers were screened using Mahalanobis distance. The results indicated that the assumption of multivariate normality was not fully satisfied (see Supplementary Table S5). Eight cases with p1 values below 0.001 were identified as potential multivariate outliers and removed before CFA estimation (see Supplementary Table S6), resulting in a final CFA sample of 204 cases. Although the multivariate normality results were less than ideal, the item-level distributions remained within acceptable ranges and the model continued to demonstrate acceptable approximate fit across the major fit indices. Accordingly, the CFA results were retained but interpreted with appropriate caution as evidence of acceptable internal structure rather than exact model fit.

Following conventional SEM reporting guidelines, χ^2^/df values below 3 are generally regarded as indicative of good fit; SRMR values below 0.08 indicate acceptable fit; and CFI, TLI, IFI, and NFI values above 0.90 indicate acceptable fit, whereas values above 0.95 indicate excellent fit (Hu & Bentler, 1999; Kline, 2023; Schermelleh-Engel et al., 2003). The two-factor model showed acceptable approximate fit overall, although RMSEA was slightly above the conventional 0.08 cutoff. Specifically, χ^2^/df was 2.538, SRMR was 0.044, and the incremental fit indices exceeded 0.90 (NFI = 0.972, IFI = 0.983, CFI = 0.983, TLI = 0.970). RMSEA was 0.088, suggesting that the model fit should be interpreted cautiously rather than as uniformly strong. Taken together, the fit indices support the adequacy of the proposed two-factor measurement model as an acceptable approximate representation of the data.

To further examine the structural validity of the scale, a competing one-factor model was also tested. As reported in Table 7, the two-factor model clearly outperformed the one-factor model across all fit indices. Several fit indices for the one-factor model failed to meet commonly accepted standards. This comparison provides further evidence that excellence-driven behavior among faculty and staff in public universities is better represented as a two-dimensional construct.

### 4.6. Reliability and Validity

Reliability and validity were assessed using the full sample of 424 cases in order to examine the internal consistency and construct validity of the final scale.

#### 4.6.1. Reliability

Internal consistency reliability was first assessed using Cronbach’s α. In general, α values above 0.60 indicate acceptable reliability, whereas values above 0.70 or 0.80 indicate good reliability (Nunnally, 1978). In the present study, deleting any item did not improve the reliability of the scale. The Cronbach’s α coefficient for the overall scale was 0.95, while the coefficients for Excellence-Driven Cognition and Learning and Excellence-Driven Display were 0.96 and 0.93, respectively. These results indicate excellent internal consistency.

Composite reliability (CR) was also examined. As shown in Table 8, the CR values were 0.947 for Excellence-Driven Cognition and Learning and 0.921 for Excellence-Driven Display, both exceeding the recommended threshold of 0.70. Taken together, the Cronbach’s α and CR results demonstrate that the scale has strong reliability.

As a supplementary indicator of reliability, split-half reliability was also examined. Because the overall scale contained 11 items and the two halves were of unequal length, the unequal-length Spearman–Brown coefficient was reported for the full scale. The resulting coefficient was 0.755, indicating acceptable split-half reliability. At the dimensional level, the Spearman–Brown coefficients were 0.952 for Excellence-Driven Cognition and Learning and 0.919 for Excellence-Driven Display, suggesting satisfactory consistency across split forms. Detailed split-half reliability results are reported in Supplementary Tables S10–S12.

#### 4.6.2. Convergent and Discriminant Validity

Convergent validity was assessed using average variance extracted (AVE), standardized factor loadings, and model fit indices. According to Fornell and Larcker (1981), AVE values above 0.50 indicate that the latent construct explains more than half of the variance in its indicators. In this study, the AVE values were 0.749 for Excellence-Driven Cognition and Learning and 0.701 for Excellence-Driven Display, both exceeding the recommended cutoff. In addition, the standardized factor loadings of the items on their respective factors ranged from 0.76 to 0.93. Together with the satisfactory CFA fit indices, these findings indicate good convergent validity.

Discriminant validity was first assessed by comparing the AVE values with the squared correlation between the two latent factors. As shown in Table 8, the AVE values for both factors were greater than the squared inter-factor correlation (φ^2^ = 0.360), satisfying the Fornell–Larcker criterion. To supplement this approach, the heterotrait–monotrait ratio (HTMT) was also examined. The HTMT value between the two dimensions was 0.621, which was below commonly recommended threshold values, thereby providing additional evidence of discriminant validity. The detailed HTMT matrix is reported in Supplementary Table S9. For transparency and readability, Table 8 includes shortened item wording alongside item codes, and the corresponding full item wording is provided in Table 3.

Overall, the findings indicate that the scale provides encouraging evidence of internal structure, internal consistency, convergent validity, discriminant validity between the two dimensions, and preliminary criterion-related validity. At the same time, these results should be interpreted as initial psychometric support rather than as a complete validation of the instrument, because temporal stability, measurement invariance, and broader empirical distinction from adjacent constructs remain to be examined.

#### 4.6.3. Criterion-Related Validity

Criterion-related validity was further examined using proactive behavior as an external construct. Proactive behavior was selected because it is theoretically related to excellence-driven behavior, in that both involve self-initiated and improvement-oriented work behavior, while remaining conceptually distinct. As shown in Supplementary Table S15, proactive behavior was positively associated with the overall excellence-driven behavior score and with both of its dimensions. In addition, the regression results reported in Supplementary Table S16 showed that excellence-driven behavior displayed significant positive associations with proactive behavior. Together, these findings provide useful preliminary evidence of criterion-related validity, although broader validation against additional external and conceptually adjacent constructs remains necessary.

## 5. Discussion

This study developed and provided initial psychometric evidence for a context-sensitive measure of excellence-driven behavior among faculty and staff in public universities through a mixed-methods measurement-development process.

### 5.1. Main Findings

The present study developed and validated a new assessment tool for measuring excellence-driven behavior among faculty and staff in public universities. Following established psychometric principles, the study integrated theoretical deduction, contextual definition, qualitative exploration, item refinement, and quantitative validation into a coherent mixed-methods measurement-development process. On the basis of literature review and logical deduction, excellence-driven behavior was first conceptually specified in the public university context. Semi-structured interviews and open-ended questionnaires were then used to generate and refine an initial pool of behavioral indicators. Subsequent item analysis, exploratory factor analysis, confirmatory factor analysis, and reliability and validity testing provided convergent evidence for the final scale structure.

The results support a two-dimensional model consisting of Excellence-Driven Cognition and Learning and Excellence-Driven Display. The first dimension captures the clarification of excellence standards, the acquisition of relevant knowledge and skills, and the internalization of pathways to excellence. The second dimension reflects the behavioral enactment of excellence through visible performance, recognition, sharing, and contribution to others.

The final 11-item instrument demonstrated satisfactory discriminant power, a clear factor structure, strong internal consistency, and acceptable convergent and discriminant validity. Taken together, these findings indicate that excellence-driven behavior can be conceptualized as a context-sensitive and measurable behavioral construct in public universities and that the present scale provides initial psychometric support for future empirical research. However, this support should be interpreted within the limits of the present design, which relied on self-reported data from a geographically restricted, non-probability sample.

Beyond confirming the psychometric adequacy of the scale, the findings also help clarify the internal logic of the construct. The two-dimensional structure suggests that excellence-driven behavior in public universities is not limited to either learning-oriented preparation or visible performance alone. Rather, it reflects a connected process in which organizational members first clarify standards, expectations, and pathways to excellence, and then translate acquired knowledge and capabilities into recognized, value-consistent contributions. In this sense, the results support a process-based understanding of excellence-driven behavior and show why a context-sensitive structure is necessary in formal institutional settings such as public universities.

### 5.2. Theoretical and Methodological Contributions

This study makes contributions at both the substantive and methodological levels. Substantively, it advances Excellence-Driven theory by shifting the focus from evaluation systems and management logic to the behavioral manifestation of excellence in formal organizational settings. Prior research has largely emphasized how Excellence-Driven is evaluated or managed, whereas the present study conceptualizes excellence-driven behavior as an independent empirical construct and clarifies its dimensional structure in the context of public universities. In doing so, it expands the theoretical reach of Excellence-Driven theory and strengthens its relevance for organizational behavior research.

The present findings also extend prior empirical work on excellence-driven behavior by moving beyond the virtual community context examined by Hou et al. (2018). Whereas the earlier scale emphasized platform-based exploratory and display behaviors embedded in online interaction, the current scale captures a more institutionally grounded form of excellence-driven behavior. In public universities, excellence is not enacted primarily through online participation cues, but through the clarification of formal standards, the acquisition of relevant capabilities, and the visible enactment of competence in performance, advancement, sharing, and contribution. The two-dimensional structure identified here therefore represents not merely a contextual replication of prior work, but a theoretically meaningful refinement of the construct for formal organizational settings.

A more critical comparison with adjacent constructs also helps clarify the distinctiveness of the present scale. Although excellence-driven behavior is positively oriented and shares certain features with proactive behavior, organizational citizenship behavior, and learning behavior, the present findings suggest that it should not be reduced to any one of these constructs alone. Compared with proactive behavior, excellence-driven behavior places stronger emphasis on clarifying organization-specific standards of excellence and aligning self-development with formal evaluation and advancement criteria. Compared with organizational citizenship behavior, its core is not discretionary helping per se, even though helping may appear as one behavioral expression within the display dimension. Compared with learning behavior, learning is not treated as an end in itself, but as part of a broader pathway through which acquired knowledge and skills are converted into visible, recognized, and value-consistent contributions. In this respect, the present two-dimensional structure provides empirical support for the conceptual distinction proposed earlier in the manuscript. The supplementary positive association with proactive behavior further supports this interpretation: excellence-driven behavior is meaningfully related to proactive action, yet its content and dimensional structure indicate a more context-bound and institutionally aligned behavioral process.

The study also contributes to behavioral science methodology by demonstrating how a new construct can be developed through meaningful integration of qualitative and quantitative methods. Rather than relying on a single analytic approach, the research combined logical deduction, qualitative evidence, expert review, pilot refinement, item analysis, and factor validation into a multi-stage measurement-development process. This design improved the contextual grounding of the scale and provided multi-step evidence supporting the internal structure and initial construct validity of the final instrument. In this respect, the study provides a concrete example of mixed-methods methodological integration in psychology and education.

A further contribution lies in the development of a context-sensitive behavioral assessment tool. Compared with prior measurement work conducted in narrower settings, such as virtual communities, the present scale is more suitable for formal organizational environments and therefore offers an initial empirical basis for future research, subject to further validation across additional samples, constructs, and testing conditions. In this sense, the study contributes not only to Excellence-Driven theory but also to the broader literature on construct development, psychometric validation, and measurement innovation in behavioral science. In addition, the positive associations observed between excellence-driven behavior and proactive behavior provide preliminary criterion-related validity evidence, suggesting that the present scale is meaningfully related to a theoretically adjacent form of positive work behavior while still capturing a more context-specific construct.

### 5.3. Practical Implications

The present findings also have practical implications for organizational management and staff development in public universities. Because the scale captures both a cognitive-learning dimension and a behavioral-display dimension, it may be used not only to measure excellence-driven behavior but also to diagnose where support is needed within departments or institutions. For example, lower levels of Excellence-Driven Cognition and Learning may indicate that faculty and staff lack clarity regarding organizational expectations, promotion pathways, or the knowledge and skills needed for development. Lower levels of Excellence-Driven Display may indicate difficulties in translating acquired capabilities into visible performance, collegial contribution, or recognized achievement.

Accordingly, the scale may support practice in at least four ways. First, it may be used as a diagnostic tool for assessing whether an institution fosters an excellence-oriented climate. Second, it may inform training and development programs by indicating whether the primary need lies in standard clarification, capability building, or behavioral enactment. Third, it may help administrators design more targeted incentive systems that encourage not only achievement but also learning, knowledge sharing, and constructive contribution to colleagues. Fourth, it may support leadership and governance practices aimed at aligning individual development with institutional goals. In this sense, the study offers practical value not only for understanding behavior but also for designing evidence-informed organizational interventions.

### 5.4. Limitations and Future Research

Despite its contributions, this study has several limitations. First, the present study relied on a geographically restricted, non-probability sample drawn from public universities in Liaoning Province, China. Although this context was theoretically meaningful for construct development, the sample composition limits the generalizability of the findings to other institutional, regional, or cultural settings. Future research should therefore examine the scale in broader and more diverse samples.

Second, although the item-level distributions were generally acceptable, the data did not fully satisfy the assumption of multivariate normality. This means that the CFA findings should be interpreted as evidence of acceptable approximate model fit and internal structure rather than as evidence of exact model fit under ideal distributional conditions. Although multivariate outliers were screened and removed prior to model estimation, future research should continue to examine the scale using additional samples and, where appropriate, estimation strategies that are more robust to multivariate non-normality.

Third, the quantitative data were based primarily on self-reports. Although several procedural remedies were adopted to reduce common method bias, such as anonymity protection, psychological separation in questionnaire design, and the avoidance of evaluative wording, these procedures cannot fully eliminate method-related inflation (Podsakoff et al., 2003). The relationships reported in the present study should therefore be interpreted as cautious evidence of association rather than as proof that method bias is absent. Future studies may combine self-reports with supervisor ratings, peer ratings, or behavioral indicators where feasible (Ng & Feldman, 2012), and may adopt longer time intervals in time-lagged designs (Weng & McElroy, 2012).

Fourth, the present study does not support strong causal interpretation. The current analyses provide evidence regarding the dimensional structure and psychometric properties of the scale, but they do not establish causal relationships among variables. Accordingly, findings involving associations between excellence-driven behavior and other constructs should be interpreted as correlational rather than causal. Future research may use longitudinal, experimental, or multi-source designs to provide stronger evidence regarding temporal ordering and causal mechanisms.

Fifth, an additional limitation concerns the scope of the validity evidence provided in the present study. The current analyses offer evidence of internal structure, internal consistency, convergent validity, discriminant validity between the two dimensions, and preliminary criterion-related validity through the supplementary proactive behavior analysis. Although the supplementary proactive behavior analysis provided useful criterion-related validity evidence, future research may strengthen this aspect of validation by examining additional external constructs and larger independent samples. However, the present study does not yet establish temporal stability, measurement invariance across groups, or broader empirical discriminant validity against adjacent constructs such as organizational citizenship behavior and learning behavior. Accordingly, the present findings should be interpreted as initial psychometric support for the scale rather than as a complete validation of all aspects of construct validity. Future research should therefore examine test–retest reliability, measurement invariance, and the empirical boundaries of the construct in relation to additional theoretically similar behaviors. In addition, intraclass correlation coefficients were not examined in the present study because such indices are more commonly used in repeated-measures or temporal stability contexts. Future research may therefore evaluate ICCs together with test–retest reliability when repeated observations are available.

Sixth, the present validation was conducted in public universities only. Although this context is theoretically meaningful, organizational culture, leadership style, and institutional environment may shape how excellence-driven behavior is expressed. Future research should test the scale in other organizational settings, including different types of universities and other formal institutions, in order to examine the boundary conditions and broader applicability of the construct.

Finally, because the scale was developed and validated in Chinese and the English item wording was prepared for reporting purposes, future cross-language applications should further examine linguistic equivalence through formal translation and back-translation procedures when an independently administered English version is developed. More broadly, future work may examine antecedents and consequences of excellence-driven behavior using more advanced quantitative designs, test the construct across subgroups defined by age, rank, position, disciplinary field, and institutional type, or compare its operation across educational and non-educational organizations. Such work would further strengthen the explanatory precision, empirical applicability, and methodological value of the construct in behavioral science research.

## 6. Conclusions

This study developed and initially validated an 11-item, two-dimensional scale of excellence-driven behavior among faculty and staff in Chinese public universities. The findings support a context-sensitive structure consisting of Excellence-Driven Cognition and Learning and Excellence-Driven Display. By integrating qualitative item generation with quantitative psychometric validation, the study provides initial psychometric support for a behavioral assessment tool that may be used in future research on excellence-driven behavior in public universities. At the same time, further research is needed to examine temporal stability, measurement invariance, broader external validity, and cross-context applicability.

## Figures and Tables

**Table 1:** Hierarchical Structure of Excellence-Driven Behavior: Labels, Categories, and Dimensions.

Label Content	Sub-Label	Dimension
Clarify the organization’s macro development direction	Clarify requirements	Excellence-Driven Cognition and Learning
Clarify work assessment criteria		
Clarify promotion requirements		
Explore effective ways to accomplish work	Observation and exploration	
Observe and emulate within the organization		
Explore methods for improvement		
Self-directed learning of relevant knowledge and skills	Active learning	
Seek external resources		
Willing to invest time and effort in learning		
Open to others’ suggestions	Open-minded consultation	
Willing to learn from others’ experiences		
Proactively consult outstanding colleagues and leaders		
Apply acquired knowledge and skills in practice	Apply what is learned	
Master relevant skills through learning		
Enhance personal performance	Achieve promotion	Excellence-Driven Display
Achieve performance improvement		
Attain professional title promotion		
Excel in performance assessments		
Excel in task completion		
Share personal success experiences with colleagues	Proactively share	
Assist colleagues in completing tasks	Willing to help	

**Table 2:** Content Validity Assessment and Item Refinement Process.

Stage	No. of Items	Main Evidence	Key Decisions	Outcome
Preliminary item pool	21	Item-level content validity was assessed by nine expert reviewers. I-CVI values ranged from 0.67 to 1.00 for relevance, 0.78 to 1.00 for clarity, and 0.67 to 1.00 for representativeness. The corresponding S-CVI/Ave values were 0.92, 0.90, and 0.87, respectively.	Based on expert review, 4 items were merged because of conceptual overlap, 2 items were deleted because they showed comparatively weaker relevance or representativeness, and 5 items were revised to improve wording precision and contextual appropriateness.	15-item test version
Qualitative pilot refinement	15	The pilot test involved 8 faculty and staff members from 2 public universities and focused on clarity, interpretability, and contextual appropriateness.	Based on qualitative feedback, 2 items were deleted because they were difficult to interpret, 4 items were merged into 2 because of content overlap, and 3 items were revised to improve wording precision and contextual fit.	Final 11-item scale
Final review and wording harmonization	11	The retained items were reviewed again by the proponents of Excellence-Driven theory, their team members, and relevant researchers.	Wording was harmonized to better fit the language used by faculty and staff in public universities while preserving conceptual coverage of the two dimensions.	Formal initial measurement questionnaire

Note. Detailed item-level content validity results for the preliminary 21-item pool are reported in Supplementary Table S1, and the full wording of those items is provided in Supplementary Materials Section S1. Detailed qualitative pilot refinement results for the preliminary 15-item test scale are reported in Supplementary Table S2, and the full wording of that 15-item version is provided in Supplementary Materials Section S2.

**Table 3:** Final 11 Items of the Excellence-Driven Behavior Scale.

Dimension	Item	Full Item Wording
Excellence-Driven Cognition and Learning	Item 1	I proactively seek to understand the performance assessment and year-end evaluation standards and requirements of my position.
	Item 2	I proactively seek to understand the promotion requirements and relevant policies associated with my position.
	Item 3	I actively explore ways to improve the quality of my work.
	Item 4	I am willing to invest time and effort in acquiring the knowledge and skills needed to improve my work performance.
	Item 5	I effectively apply the knowledge and skills I have acquired to my work tasks.
	Item 6	I am eager to learn from exemplary colleagues or leaders in the organization, take their suggestions seriously, and draw on their experience.
Excellence-Driven Display	Item 7	I receive praise and recognition from colleagues and leaders because of my work ability.
	Item 8	I can use the knowledge and skills I have mastered to achieve superior performance appraisal results.
	Item 9	I can leverage my knowledge and skills to attain promotion in position or professional title.
	Item 10	I often share the knowledge, experience, and methods I have acquired with my colleagues.
	Item 11	I regularly use the knowledge and skills I have acquired to help colleagues complete relevant tasks.

Note. The final scale consists of 11 items across two dimensions: six items for Excellence-Driven Cognition and Learning and five items for Excellence-Driven Display. The questionnaire used in the actual study was administered in Chinese. The English wording reported here is the publication version translated from the original Chinese items and checked for conceptual correspondence by bilingual members of the research team.

**Table 4:** Demographic Characteristics of the Full Sample and the EFA/CFA Subsamples.

Variable	Category	Full Sample (*N* = 424)	EFA Subsample (*n* = 212)	CFA Subsample (*n* = 212)
Gender	Male	189 (44.58%)	97 (45.75%)	92 (43.40%)
	Female	235 (55.42%)	115 (54.25%)	120 (56.60%)
Age	Under 25 years old	69 (16.27%)	31 (14.62%)	38 (17.92%)
	26–35 years old	211 (49.76%)	107 (50.47%)	104 (49.06%)
	36–45 years old	121 (28.54%)	62 (29.25%)	59 (27.83%)
	Over 46 years old	23 (5.42%)	12 (5.66%)	11 (5.19%)
Education level	Master	337 (79.48%)	166 (78.30%)	171 (80.66%)
	Doctorate	87 (20.52%)	46 (21.70%)	41 (19.34%)
Years of service	Less than 1 year	46 (10.84%)	19 (8.96%)	27 (12.74%)
	1–3 years	83 (19.58%)	49 (23.11%)	34 (16.04%)
	4–6 years	210 (49.53%)	97 (45.75%)	113 (53.30%)
	7–9 years	51 (12.03%)	27 (12.74%)	24 (11.32%)
	More than 10 years	34 (8.02%)	20 (9.44%)	14 (6.60%)

Note. The full sample consisted of 424 valid respondents. For factor validation, the full sample was randomly divided into an exploratory factor analysis (EFA) subsample and a confirmatory factor analysis (CFA) subsample, each containing 212 respondents. The demographic distributions of the two analytic subsamples were broadly comparable.

**Table 5:** Results of Item Discrimination and Item Analysis.

Question Item	Short Item Wording	Critical Value (t-Value)	Correlation with Total Score (r-Value)
Item 1	Understand performance and year-end evaluation standards	19.98 ***	0.86 **
Item 2	Understand promotion requirements and relevant policies	24.96 ***	0.94 **
Item 3	Explore ways to improve work quality	25.25 ***	0.95 **
Item 4	Invest time and effort in acquiring work-related knowledge and skills	24.96 ***	0.95 **
Item 5	Apply acquired knowledge and skills to work tasks	22.63 ***	0.95 **
Item 6	Learn from exemplary colleagues and leaders	20.90 ***	0.91 **
Item 7	Receive recognition for work ability	11.49 ***	0.84 **
Item 8	Use knowledge and skills to achieve strong appraisal results	13.77 ***	0.86 **
Item 9	Use knowledge and skills to attain promotion	13.13 ***	0.83 **
Item 10	Share acquired knowledge, experience, and methods with colleagues	13.62 ***	0.87 **
Item 11	Use acquired knowledge and skills to help colleagues complete tasks	14.48 ***	0.86 **

Note: *N* (high-score group) = 114, *N* (low-score group) = 114, *** *p* < 0.001, ** *p* < 0.01. The full wording of the final 11 Items is provided in Table 3.

**Table 6:** Exploratory Factor Analysis Results for the Final Scale (Direct Oblimin Rotation).

Question Item	Short Item Wording	Excellence-Driven Cognition and Learning	Excellence-Driven Display
Item 1	Understand performance and year-end evaluation standards	0.77	0.08
Item 2	Understand promotion requirements and relevant policies	0.96	−0.02
Item 3	Explore ways to improve work quality	1.00	−0.11
Item 4	Invest time and effort in acquiring work-related knowledge and skills	0.99	−0.06
Item 5	Apply acquired knowledge and skills to work tasks	0.87	0.09
Item 6	Learn from exemplary colleagues and leaders	0.78	0.12
Item 7	Receive recognition for work ability	−0.02	0.91
Item 8	Use knowledge and skills to achieve strong appraisal results	0.13	0.76
Item 9	Use knowledge and skills to attain promotion	−0.11	0.95
Item 10	Share acquired knowledge, experience, and methods with colleagues	0.08	0.86
Item 11	Use acquired knowledge and skills to help colleagues complete tasks	0.06	0.86

Note. *N* = 212. Pattern coefficients from the direct oblimin solution are reported. The rotation converged after five iterations. The correlation between the two factors was 0.61. The corresponding varimax and Promax solutions are reported in Supplementary Tables S7 and S8. The full wording of the final 11 Items is provided in Table 3.

**Table 7:** Comparison of Model Fit Indices for the One-Factor and Two-Factor Models.

Fit Index	χ^2^/df	RMSEA	SRMR	NFI	IFI	CFI	TLI
Two-Dimensional Structure	2.538	0.088	0.044	0.972	0.983	0.983	0.970
One-Dimensional Structure	8.599	0.195	0.146	0.902	0.913	0.912	0.853

Note: *N* = 204. Lower χ^2^/df, RMSEA, and SRMR values indicate better fit, whereas higher NFI, IFI, CFI, and TLI values indicate better fit.

**Table 8:** Factor Loadings, Composite Reliability, Average Variance Extracted, and Discriminant Validity.

Question Item	Short Item Wording	Factor Loadings		CR	AVE	∅^2^
		Excellence-Driven Cognition and Learning	Excellence-Driven Display			
Item 1	Understand performance and year-end evaluation standards	0.77	0.08	0.962	0.810	0.360
Item 2	Understand promotion requirements and relevant policies	0.96	−0.02			
Item 3	Explore ways to improve work quality	1.00	−0.11			
Item 4	Invest time and effort in acquiring work-related knowledge and skills	0.99	−0.06			
Item 5	Apply acquired knowledge and skills to work tasks	0.87	0.09			
Item 6	Learn from exemplary colleagues and leaders	0.78	0.12			
Item 7	Receive recognition for work ability	−0.02	0.91	0.940	0.757	0.360
Item 8	Use knowledge and skills to achieve strong appraisal results	0.13	0.76			
Item 9	Use knowledge and skills to attain promotion	−0.11	0.95			
Item 10	Share acquired knowledge, experience, and methods with colleagues	0.08	0.86			
Item 11	Use acquired knowledge and skills to help colleagues complete tasks	0.06	0.86			

Note: *N* = 424. Standardized factor loadings are reported. CR = composite reliability; AVE = average variance extracted. The full wording of the final 11 items is provided in Table 3. The HTMT value for the two dimensions is reported in Supplementary Table S9.

## Data Availability

The data that support the findings of this study are available from the corresponding author upon reasonable request. Because the dataset contains potentially sensitive information from faculty and staff in public universities, it is not publicly available. De-identified data may be shared for academic verification purposes subject to ethical and privacy considerations.

## Supplementary Materials

The following supporting information can be downloaded at: https://www.mdpi.com/article/10.3390/bs16060950/s1, Figure S1. scree plot. Figure S2. Scree Plot (R Version). Table S1: Item-Level Content Validity Results for the Preliminary 21-Item Pool. Table S2. Qualitative Pilot Refinement Results for the Preliminary 15-Item Test Scale. Table S3. Descriptive Statistics for the Final 11 Items. Table S4. Additional Distribution Indicators for the Final 11 Items. Table S5. Univariate and Multivariate Normality Assessment for the CFA Subsample. Table S6. Multivariate Outlier Screening Based on Mahalanobis Distance in the CFA Subsample. Table S7. Exploratory Factor Analysis Results for the Final Scale (Varimax Rotation). Table S8. Exploratory Factor Analysis Results for the Final Scale (Promax Rotation). Table S9. Discriminant validity-Heterotrait-monotrait ratio (HTMT)–Matrix. Table S10. Split-Half Reliability for the Overall Scale. Table S11. Split-Half Reliability for Dimension 1. Table S12. Split-Half Reliability for Dimension 2. Table S13. Extraction Communalities for the Final 11 Items in the EFA Sample. Table S14. Demographic Characteristics of the Supplementary Proactive Behavior Sample. Table S15. Correlations Between Excellence-Driven Behavior (and Its Dimensions) and Proactive Behavior. Table S16. Regression Results for Excellence-Driven Behavior (and Its Dimensions) Predicting Proactive Behavior. Table S17. Comparison Between Paper-Based and Online Respondents Across the 11 Scale Items. Table S18. Conceptual Comparison Between Excellence-Driven Behavior and Adjacent Constructs.
