# Supplementary material for: Developing and Validating a Context-Sensitive Scale of Excellence-Driven Behavior in Public Universities: A Mixed-Methods Psychometric Study
Source: Behav Sci (Basel). 2026 Jun 9;16(6):950. doi: 10.3390/bs16060950 (PMC13296322; doi:10.3390/bs16060950)
Supplement: Supplementary file 1 [file behavsci-16-00950-s001.zip › behavsci-4319824-supplementary.pdf]

Supplementary Material

Table S1. Item-Level Content Validity Results for the Preliminary 21-Item Pool

Initial Item	Short item wording	I-CVI (Relevance)	I-CVI (Clarity)	I-CVI (Representativeness)	Decision / Outcome in 15-item test pool
Item 1	Understand organizational rules and developments	0.89	0.78	0.78	Revised → Test Item 1
Item 2	Understand job-related policy requirements	0.89	0.89	0.78	Merged into policy-related items (Test Items 2–3)
Item 3	Understand promotion requirements and evaluation standards	1.00	0.89	0.89	Revised → Test Item 3
Item 4	Understand performance and year-end evaluation standards	1.00	0.89	1.00	Revised → Test Item 2
Item 5	Identify knowledge and skills required for the job	1.00	1.00	1.00	Retained → Test Item 4
Item 6	Explore ways to improve work quality	1.00	0.89	1.00	Retained → Test Item 5
Item 7	Invest time and effort in mastering work-related knowledge and skills	1.00	0.89	1.00	Retained → Test Item 6
Item 8	Participate in learning or training to improve competence	0.89	0.89	0.89	Retained → Test Item 7
Item 9	Learn from others' suggestions and experience	1.00	1.00	1.00	Retained → Test Item 8
Item 10	Apply acquired knowledge and skills	1.00	1.00	1.00	Retained → Test Item 10

Initial Item	Short item wording	I-CVI (Relevance)	I-CVI (Clarity)	I-CVI (Representativeness)	Decision / Outcome in 15-item test pool
	to actual work				
Item 11	Transform knowledge and skills into promotion-related achievements	0.89	0.89	0.89	Retained → Test Item 9
Item 12	Use knowledge and ability to achieve better appraisal results	1.00	1.00	1.00	Retained → Test Item 12
Item 13	Apply innovative ideas to actual work	0.67	0.89	0.67	Deleted
Item 14	Use knowledge and ability to perform better and gain recognition	1.00	0.89	0.89	Revised → Test Item 11
Item 15	Receive praise and recognition for performing work well	0.89	0.89	0.78	Merged into Test Item 11
Item 16	Receive recognition for strong work ability	0.89	0.89	0.78	Merged into Test Item 11
Item 17	Use knowledge and ability to attain promotion	1.00	1.00	1.00	Retained → Test Item 13
Item 18	Share ideas, experience, and methods with colleagues	1.00	0.89	0.89	Revised → Test Item 14
Item 19	Shared ideas and experience are appreciated by others	0.78	0.89	0.67	Merged into Test Item 14
Item 20	Use knowledge and ability to help colleagues complete work	1.00	1.00	1.00	Retained → Test Item 15
Item 21	Increasing recognition of my abilities in the organization	0.67	0.78	0.67	Deleted

Note. Nine expert reviewers evaluated each preliminary item in terms of relevance, clarity, and representativeness using separate four-point rating scales. Item-level content validity indices (I-CVI) were calculated as the proportion of experts assigning a rating of 3 or 4 to a given item. For a panel of nine experts, an I-CVI value of 0.78 or above was considered acceptable. Based on expert review, four items were merged because of conceptual overlap, two items were deleted because they showed comparatively weaker relevance or representativeness for the target construct, and five items were revised to improve wording precision and contextual appropriateness, resulting in the 15-item test version.

S-CVI/Ave (Relevance) = 0.92
S-CVI/Ave (Clarity) = 0.90
S-CVI/Ave (Representativeness) = 0.87

S1. Full Wording of the Preliminary 21-Item Pool

1. I proactively seek to understand the organization's rules, regulations, and latest developments.
2. I proactively seek to understand all policy requirements related to my job position.
3. I proactively seek to understand the promotion requirements and evaluation standards associated with my position.
4. I proactively seek to understand the performance and year-end evaluation standards and policy requirements associated with my position.
5. I proactively seek to understand what knowledge and skills are required for the work I perform.
6. I spend time exploring how I can complete my work more effectively.
7. I am willing to devote time and effort to learning and mastering the knowledge and skills required for my work.
8. I proactively participate in relevant learning or training activities through various channels in order to improve my professional or job-related competence.
9. I am willing to listen to suggestions from other organizational members or leaders and learn from their experience.
10. I am able to apply the knowledge and skills I have acquired through learning or training to my actual work.
11. I am able to transform the knowledge or skills I have mastered into achievements needed for professional title evaluation or promotion.
12. I am able to use my knowledge and abilities to achieve better performance appraisal results.
13. I apply my own innovative ideas to my actual work.
14. I am able to use my knowledge and abilities to perform my work better and gain recognition from colleagues and leaders.
15. I receive praise and recognition from colleagues and leaders because I perform my work well.
16. I receive praise and recognition from colleagues and leaders because of my strong work ability.

17. I am able to use my knowledge and abilities to attain promotion in professional title or job rank.
18. I am willing to share my ideas, experience, and methods with my colleagues.
19. The ideas, experience, and methods I share are often appreciated by colleagues and leaders.
20. I use my knowledge and abilities to help colleagues complete relevant work tasks.
21. Within the organization, recognition of my abilities is increasing over time.

Table S2. Qualitative Pilot Refinement Results for the Preliminary 15-Item Test Scale

Test Item	Short item wording	Clarity	Interpretability	Context appropriateness	Recommended decision	Outcome in final 11-item scale	Recommended rationale
Item 1	Monitor organizational development trends	Acceptable	Moderate concern	Moderate concern	Deleted	—	Too broad and weakly tied to the core behavioral process of excellence-driven behavior in the job context.
Item 2	Understand performance and year-end evaluation standards	Acceptable	Acceptable	Minor concern	Revised	Final CL Item 1	Wording refined to better align with "performance assessment and year-end evaluation standards and requirements."
Item 3	Understand promotion requirements and related policies	Acceptable	Acceptable	Acceptable	Retained	Final CL Item 2	Clear and contextually appropriate; directly reflects promotion-related cognitive orientation.
Item 4	Identify knowledge and skills required for the job	Acceptable	Acceptable	Acceptable	Merged	Merged with Item 6 → Final CL Item 4	Conceptually overlaps with learning/mastery content in Item 6.

Test Item	Short item wording	Clarity	Interpretability	Context appropriateness	Recommended decision	Outcome in final 11-item scale	Recommended rationale
Item 5	Explore ways to improve work quality	Minor concern	Acceptable	Acceptable	Revised	Final CL Item 3	Wording refined from “complete work more effectively” to “improve the quality of my work” for better precision.
Item 6	Invest time and effort in mastering work-related knowledge and skills	Acceptable	Acceptable	Acceptable	Merged	Merged with Item 4 → Final CL Item 4	Combined with Item 4 to form a more complete cognition-to-learning statement.
Item 7	Participate in learning or training to improve competence	Acceptable	Acceptable	Minor concern	Merged	Merged with Item 8 → Final CL Item 6	Procedural learning/training content overlapped with learning from others.
Item 8	Learn from others' suggestions and experience	Acceptable	Acceptable	Acceptable	Merged	Merged with Item 7 → Final CL Item 6	Combined with Item 7 to create a more contextually rich learning-from-others item.
Item 9	Transform knowledge and skills into promotion-related achievements	Moderate concern	Major concern	Minor concern	Deleted	—	Considered difficult to interpret because of abstract wording and mixed outcome references.
Item	Apply acquired	Acceptable	Acceptable	Acceptable	Retained	Final CL	Clear behavioral application item with

Test Item	Short item wording	Clarity	Interpretability	Context appropriateness	Recommended decision	Outcome in final 11-item scale	Recommended rationale
10	knowledge and skills to actual work					Item 5	strong contextual fit.
Item 11	Use knowledge and ability to perform better and gain recognition	Minor concern	Acceptable	Acceptable	Revised	Final D Item 1	Refined to focus more directly on recognition for work ability rather than a mixed performance-plus-recognition expression.
Item 12	Use knowledge and ability to achieve better appraisal results	Acceptable	Acceptable	Acceptable	Retained	Final D Item 2	Clear and directly linked to performance appraisal outcomes.
Item 13	Use knowledge and ability to attain promotion	Acceptable	Acceptable	Acceptable	Retained	Final D Item 3	Clear and contextually appropriate promotion-related display item.
Item 14	Share experience and methods with colleagues	Acceptable	Acceptable	Acceptable	Retained	Final D Item 4	Retained as a clear collegial sharing behavior; later wording harmonization did not constitute a separate pilot-triggered revision.
Item 15	Use knowledge and ability to help colleagues complete	Acceptable	Acceptable	Acceptable	Retained	Final D Item 5	Clear helping behavior and strong fit with the display dimension.

Test Item	Short item wording	Clarity	Interpretability	Context appropriateness	Recommended decision	Outcome in final 11-item scale	Recommended rationale
	work						

Note. The pilot test served as a qualitative refinement step rather than a formal psychometric test. Feedback from eight faculty and staff members from two public universities was used to assess item clarity, interpretability, and contextual appropriateness. Based on this feedback, two items were removed because they were considered difficult to interpret, four items were merged into two due to content overlap, and three items were revised to improve wording precision and contextual fit.

S2. Full Wording of the Preliminary 15-Item Test Scale

1. I pay attention to the latest developments in the organization.
2. I proactively seek to understand the performance and year-end evaluation standards and policy requirements associated with my position.
3. I proactively seek to understand the promotion requirements and related policies associated with my position.
4. I proactively seek to understand what knowledge and skills are required for the work I perform.
5. I spend time exploring how I can complete my work more effectively.
6. I am willing to devote time and effort to learning and mastering the knowledge and skills required for my work.
7. I proactively participate in relevant learning or training activities in order to improve my professional or job-related competence.
8. I am willing to listen to suggestions from other organizational members or leaders and learn from their experience.
9. I am able to transform the knowledge or skills I have mastered into achievements needed for professional title evaluation or promotion.
10. I am able to apply the knowledge and skills I have acquired through learning or training to my actual work.
11. I am able to use my knowledge and abilities to perform my work better and gain recognition from colleagues and leaders.
12. I am able to use my knowledge and abilities to achieve better performance appraisal results.
13. I am able to use my knowledge and abilities to attain promotion in professional title or job rank.
14. I am willing to share my experience and methods with my colleagues.

15. I use my knowledge and abilities to help colleagues complete relevant work tasks.

Table S3. Descriptive Statistics for the Final 11 Items

Item	N	Min	Max	M	SD	Variance	Skewness	Kurtosis
Item 1	424	1	5	3.21	0.923	0.852	0.115	0.028
Item 2	424	1	5	3.27	0.900	0.811	-0.023	0.235
Item 3	424	1	5	3.48	1.056	1.115	0.026	-0.560
Item 4	424	1	5	3.34	0.952	0.906	0.053	0.005
Item 5	424	1	5	3.25	0.843	0.710	-0.132	0.612
Item 6	424	1	5	3.51	0.987	0.974	-0.144	-0.165
Item 7	424	1	5	4.36	0.861	0.742	-1.571	2.446
Item 8	424	2	5	4.19	0.807	0.651	-0.957	0.686
Item 9	424	2	5	4.27	0.802	0.643	-0.944	0.363
Item 10	424	1	5	4.41	0.829	0.687	-1.446	1.617
Item 11	424	1	5	4.11	0.943	0.889	-1.182	1.176

Note. M = mean; SD = standard deviation; Min = minimum; Max = maximum. Item-level skewness and kurtosis values were within acceptable ranges for preliminary psychometric screening.

Table S4. Additional Distribution Indicators for the Final 11 Items

Item	M	SD	SE	95% CI for M	Skewness	Kurtosis
Item 1	3.21	0.923	0.045	[3.12, 3.30]	0.115	0.028
Item 2	3.27	0.900	0.044	[3.18, 3.35]	-0.023	0.235
Item 3	3.48	1.056	0.051	[3.38, 3.58]	0.026	-0.560
Item 4	3.34	0.952	0.046	[3.25, 3.43]	0.053	0.005
Item 5	3.25	0.843	0.041	[3.17, 3.33]	-0.132	0.612
Item 6	3.51	0.987	0.048	[3.42, 3.60]	-0.144	-0.165
Item 7	4.36	0.861	0.042	[4.28, 4.44]	-1.571	2.446
Item 8	4.19	0.807	0.039	[4.11, 4.27]	-0.957	0.686
Item 9	4.27	0.802	0.039	[4.19, 4.35]	-0.944	0.363
Item 10	4.41	0.829	0.040	[4.33, 4.49]	-1.446	1.617
Item 11	4.11	0.943	0.046	[4.02, 4.20]	-1.182	1.176

Note. M = mean; SD = standard deviation; SE = standard error; CI = confidence interval. This table presents additional distribution indicators for the final 11 items in a publication-ready format. The item-level descriptive statistics indicate that the observed distributions were generally acceptable for

psychometric analysis, although several display-related items showed moderate negative skewness, consistent with relatively high endorsement levels.

Table S5. Univariate and Multivariate Normality Assessment for the CFA Subsample

Variable	min	max	skew	c.r.	kurtosis	c.r.
Item 11	1.000	5.000	-1.161	-6.721	1.116	3.230
Item 10	1.000	5.000	-1.498	-8.670	1.776	5.139
Item 9	2.000	5.000	-.905	-5.237	.156	.450
Item 8	2.000	5.000	-.941	-5.445	.519	1.502
Item 7	1.000	5.000	-1.495	-8.651	2.240	6.483
Item 6	1.000	5.000	-.295	-1.708	-.112	-.323
Item 5	1.000	5.000	-.196	-1.133	.715	2.070
Item 4	1.000	5.000	.015	.085	.029	.084
Item 3	1.000	5.000	.048	.279	-.508	-1.470
Item 2	1.000	5.000	-.013	-.072	.098	.282
Item 1	1.000	5.000	.064	.371	-.004	-.012
Multivariate					35.772	14.994

Table S6. Multivariate Outlier Screening Based on Mahalanobis Distance in the CFA Subsample

Observation number	Mahalanobis d-squared	p1	p2
143	55.382	.000	.000
126	42.695	.000	.000
28	41.240	.000	.000
211	38.224	.000	.000
139	38.170	.000	.000
127	36.977	.000	.000
145	35.124	.000	.000
68	34.161	.000	.000
56	32.826	.001	.000
144	32.826	.001	.000
5	30.334	.001	.000
6	29.115	.002	.000
181	27.027	.005	.000
192	26.734	.005	.000
41	26.725	.005	.000
180	25.448	.008	.000
212	24.711	.010	.000
177	23.658	.014	.000
133	22.987	.018	.000

Observation number	Mahalanobis d-squared	p1	p2
128	21.285	.031	.000
24	20.763	.036	.000
32	20.744	.036	.000
138	19.586	.051	.001
135	19.482	.053	.000
33	19.209	.057	.001
191	17.821	.086	.042
173	17.368	.097	.092
95	17.076	.106	.129
18	16.828	.113	.163
54	16.716	.117	.153
108	16.689	.117	.118
124	16.463	.125	.147
132	16.310	.130	.157
189	15.914	.144	.280
184	15.807	.148	.274
8	15.433	.164	.430
9	15.433	.164	.360
10	15.433	.164	.294
204	15.088	.178	.445
43	15.057	.180	.397
130	15.030	.181	.348
168	14.979	.183	.317
179	14.979	.183	.258
81	14.638	.200	.414
209	14.614	.201	.365
197	14.594	.202	.317
205	14.594	.202	.259
206	14.594	.202	.208
210	14.594	.202	.164
60	14.367	.213	.235
196	14.304	.217	.221
198	14.294	.217	.181
203	14.294	.217	.141
186	14.259	.219	.121
188	13.944	.236	.234
85	13.879	.240	.225
137	13.756	.247	.250
141	13.756	.247	.203

Observation number	Mahalanobis d-squared	p1	p2
142	13.756	.247	.162
13	13.682	.251	.160
15	13.682	.251	.125
207	13.644	.253	.110
20	13.531	.260	.125
39	13.469	.264	.120
40	13.469	.264	.092
140	13.248	.277	.152
92	12.599	.320	.579
182	12.497	.327	.608
106	12.491	.328	.556
1	12.181	.350	.752
2	12.181	.350	.703
3	12.181	.350	.651
4	12.181	.350	.597
96	11.961	.367	.725
23	11.824	.377	.778
31	11.824	.377	.733
183	11.543	.399	.872
7	11.405	.410	.906
202	11.383	.412	.891
201	11.380	.412	.863
174	11.304	.418	.872
136	11.234	.424	.878
17	11.032	.441	.935
199	10.841	.457	.967
123	10.756	.464	.972
148	10.683	.470	.975
195	10.681	.470	.966
208	10.681	.470	.954
82	10.612	.476	.957
101	10.612	.476	.943
80	10.521	.484	.953
98	10.521	.484	.938
176	10.134	.518	.992
84	10.012	.529	.995
90	9.910	.538	.997
27	9.684	.559	.999
38	9.603	.566	.999

Observation number	Mahalanobis d-squared	p1	p2
193	9.494	.576	1.000
194	9.494	.576	.999
200	9.494	.576	.999

Note. Cases with p1 < 0.001 were treated as potential multivariate outliers; eight cases met this criterion and were excluded before CFA estimation, resulting in a final CFA sample of 204 cases.

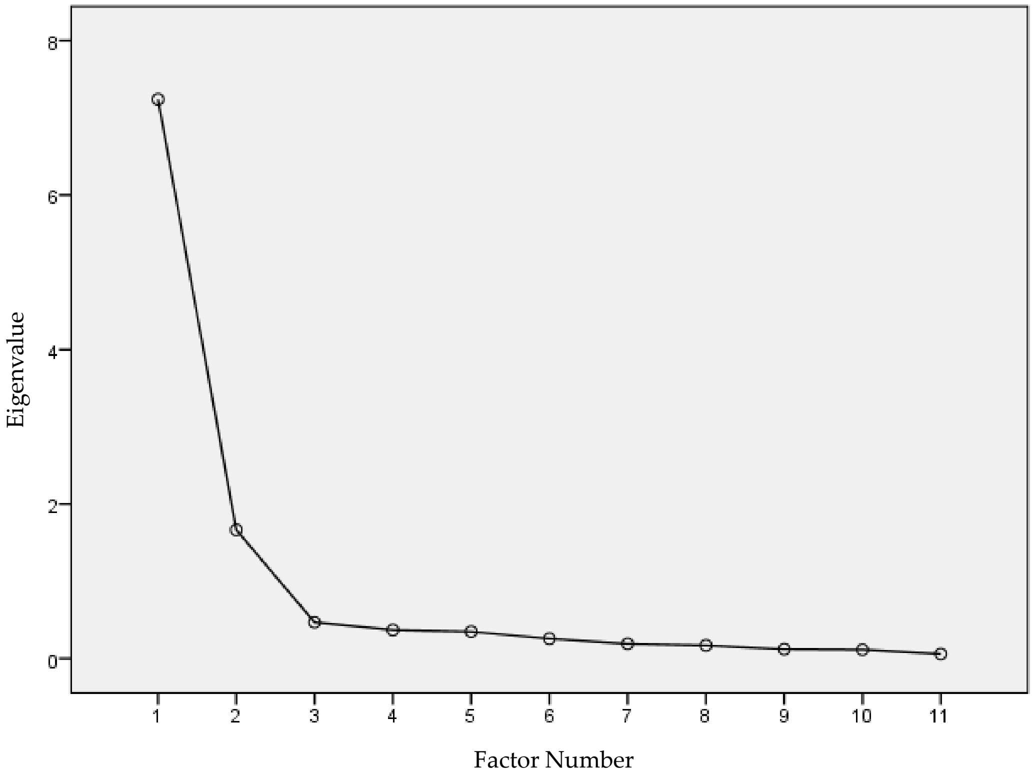

Figure S1. scree plot

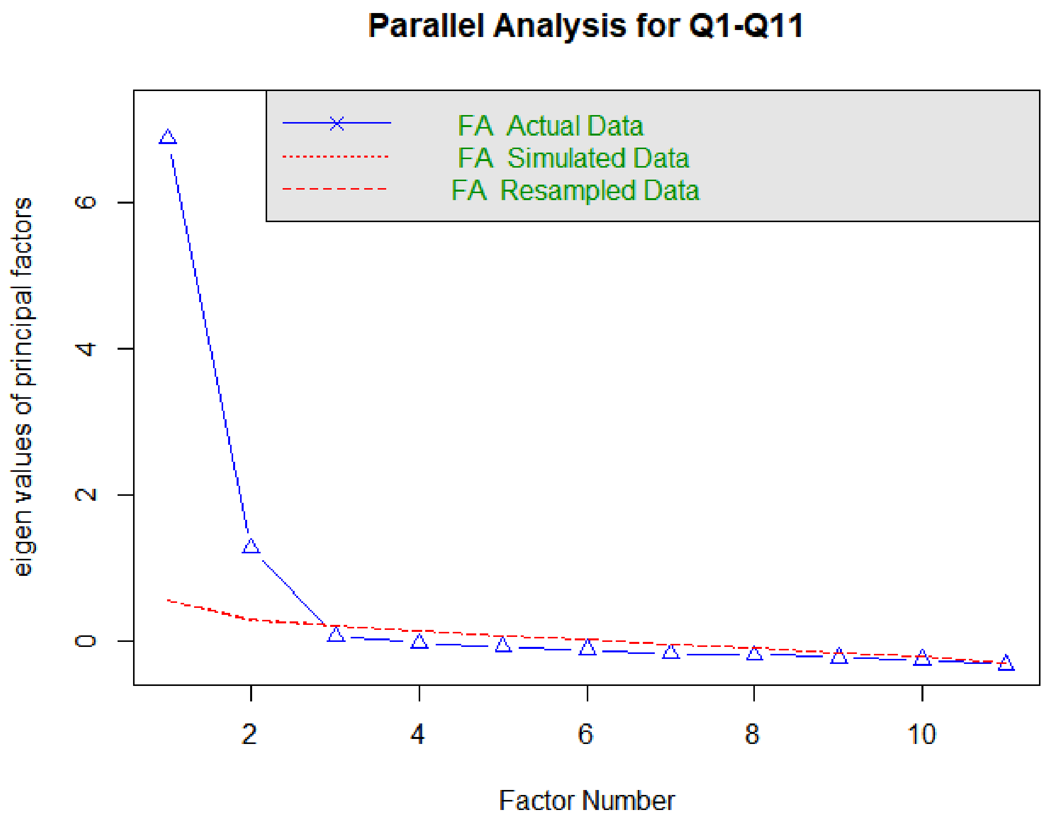

Figure S2. Scree Plot (R Version)

S3. Parallel Analysis Results

To provide additional support for factor retention beyond the Kaiser criterion, a parallel analysis was conducted in R. The results indicated that two factors should be retained. This conclusion was consistent with the SPSS scree plot (**Figure S1**), the R-based scree plot (**Figure S2**), and the substantively similar factor solutions obtained under direct oblimin and Promax rotation.

The parallel analysis therefore provided convergent evidence for the two-factor structure of the scale. In substantive terms, the results supported the distinction between Excellence-Driven Cognition and Learning and Excellence-Driven Display, which was also consistent with the qualitative findings and the theoretically derived process logic of the construct.

Summary of Parallel Analysis and Oblique Rotation Results

Analysis Component	Result
Parallel analysis conclusion	Two factors retained
Rotation methods examined	Promax and direct oblimin
Substantive consistency of solutions	Both supported the same two-factor structure
Factor correlation (Promax)	0.64
Factor correlation (Direct Oblimin)	0.61

Analysis Component	Result
RMSR	0.02
TLI	0.935
RMSEA	0.117
BIC	-48.91

Note. The parallel analysis supported a two-factor solution. The Promax and direct oblimin solutions yielded substantively similar loading patterns, and both confirmed that the two latent dimensions were moderately correlated rather than independent. These findings provided additional justification for reporting the direct oblimin solution as the primary EFA result in the main manuscript, while retaining the varimax and Promax solutions in **Supplementary Tables S7 and S8** as robustness checks.

Table S7. Exploratory Factor Analysis Results for the Final Scale (Varimax Rotation)

Question Item	Excellence-Driven Cognition and Learning	Excellence-Driven Display
Item 1	**0.75**	0.32
Item 2	**0.91**	0.29
Item 3	**0.92**	0.22
Item 4	**0.92**	0.26
Item 5	**0.86**	0.37
Item 6	**0.78**	0.36
Item 7	0.26	**0.86**
Item 8	0.36	**0.77**
Item 9	0.19	**0.87**
Item 10	0.34	**0.84**
Item 11	0.32	**0.83**
Variance Explained	44.31%	36.64%

Note: N = 212. The rotation method for exploratory factor analysis is the varimax rotation method.

Table S8. Exploratory Factor Analysis Results for the Final Scale (Promax Rotation)

Question Item	Excellence-Driven Cognition and Learning	Excellence-Driven Display
Item 1	0.76	0.09
Item 2	0.96	-0.01
Item 3	1.00	-0.10
Item 4	0.99	-0.05
Item 5	0.87	0.10
Item 6	0.78	0.12
Item 7	-0.03	0.91
Item 8	0.13	0.76
Item 9	-0.11	0.95
Item 10	0.08	0.86
Item 11	0.05	0.86

Note. N = 212. Pattern coefficients from the Promax solution are

reported. The rotation converged after three iterations.

Table S9. Discriminant validity - Heterotrait-monotrait ratio (HTMT) - Matrix

	Dimension 1	Dimension 2
Dimension 1		
Dimension 2	0.621	

Table S10. Split-Half Reliability for the Overall Scale

Cronbach's Alpha	Part 1	Value	.957
		Number of items	6[a]
	Part 2	Value	.930
		Number of items	5[b]
	Total number of items		11
Correlation between forms			.605
Spearman-Brown Coefficient	Equal length		.754
	Unequal length		.755
Guttman Split-Half Coefficient			.731
Items: Item 1, Item 2, Item 3, Item 4, Item 5, Item 6.a			
Items: Item 7, Item 8, Item 9, Item 10, Item 11.b			

Table S11. Split-Half Reliability for Dimension 1

Cronbach's Alpha	Part 1	Value	.915
		Number of items	3[a]
	Part 2	Value	.925
		Number of items	3[b]
	Total number of items		6
Correlation between forms			.909
Spearman-Brown Coefficient	Equal length		.952
	Unequal length		.952
Guttman Split-Half Coefficient			.952
Items: Item 1, Item 2, Item 3.a			
Items: Item 4, Item 5, Item 6.b			

Table S12. Split-Half Reliability for Dimension 2

Cronbach's Alpha	Part 1	Value	.880
		Number of items	3[a]
	Part 2	Value	.892
		Number of items	2[b]
	Total number of items		5
Correlation between forms			.845
Spearman-Brown Coefficient	Equal length		.916

	Unequal length	.919
Guttman Split-Half Coefficient		.898
Items: Item 7, Item 8, Item 9		
Items: Item 10, Item 11.b		

Table S13. Extraction Communalities for the Final 11 Items in the EFA Sample

Item	Initial	Extraction
Item 1	1.000	.669
Item 2	1.000	.909
Item 3	1.000	.900
Item 4	1.000	.913
Item 5	1.000	.865
Item 6	1.000	.730
Item 7	1.000	.802
Item 8	1.000	.714
Item 9	1.000	.792
Item 10	1.000	.815
Item 11	1.000	.796

Table S14. Demographic Characteristics of the Supplementary Proactive Behavior Sample

Variable	Content	Number	Percentage (%)
Gender	Male	47	44.76%
	Female	58	55.24%
Age	Under 25 years old	17	16.19%
	26-35 years old	53	50.48%
	36-45 years old	31	29.52%
	Over 46 years old	4	3.81%
Education level	Master	82	78.10%
	Doctorate	23	21.90%
Years of service	Less than 1 year	11	10.47%
	1-3 years	20	19.05%
	4-6 years	54	51.43%

7-9 years	13	12.38%
More than 10 years	7	6.67%

Table S15. Correlations Between Excellence-Driven Behavior (and Its Dimensions) and Proactive Behavior

	M	SD	EDB	CL	D	PB
EDB	8.59	3.00	1	.403**	.361**	.300**
CL	3.34	0.83	.403**	1	.599**	.636**
D	4.29	0.71	.361**	.599**	1	.491**
PB	3.73	0.66	.300**	.636**	.491**	1

Note. EDB = overall excellence-driven behavior; CL = Excellence-Driven Cognition and Learning; D = Excellence-Driven Display; PB = proactive behavior. ** Correlation is significant at the 0.01 level (2-tailed).

Table S16. Regression Results for Excellence-Driven Behavior (and Its Dimensions) Predicting Proactive Behavior

Variables	PB	
	B	SE
EDB	.066**	.021

Note. EDB = overall excellence-driven behavior; PB = proactive behavior.
N = 105. *p < 0.05, **p < 0.01, ***p < 0.001.

Table S17. Comparison Between Paper-Based and Online Respondents Across the 11 Scale Items

**Item**	**Online M (SD)**	**Paper M (SD)**	**Levene p**	**t**	**df**	**p**	**Mean diff.**
Item 1	3.19 (0.94)	3.22 (0.918)	0.996	-0.229	422	0.819	-0.023
Item 2	3.24 (0.934)	3.28 (0.889)	0.558	-0.411	422	0.681	-0.041
Item 3	3.52 (1.091)	3.46 (1.044)	0.381	0.458	422	0.647	0.053
Item 4	3.3 (0.968)	3.35 (0.947)	0.884	-0.542	422	0.588	-0.057
Item 5	3.17 (0.841)	3.27 (0.843)	0.531	-1.165	422	0.245	-0.108
Item 6	3.49 (0.998)	3.52 (0.984)	0.938	-0.26	422	0.795	-0.028
Item 7	4.32 (0.876)	4.38 (0.857)	0.868	-0.653	422	0.514	-0.062
Item 8	4.18 (0.823)	4.2 (0.802)	0.929	-0.241	422	0.81	-0.021
Item 9	4.28 (0.804)	4.27 (0.802)	0.764	0.147	422	0.883	0.013
Item 10	4.37 (0.844)	4.43 (0.824)	0.537	-0.667	422	0.505	-0.061
Item 11	4.09 (1.001)	4.11 (0.923)	0.346	-0.244	422	0.808	-0.025

Note. Online M (SD) and Paper M (SD) report item means and standard deviations by response mode. Levene p reports the test of equality of variances; p reports the two-tailed t-test for mean differences.

Table S18. Conceptual Comparison Between Excellence-Driven Behavior and Adjacent Constructs

Dimension	EDB	Proactive Behavior	OCB	Learning Behavior
Core orientation	Clarifying standards of excellence, developing relevant capabilities, and translating them into visible, value-consistent contributions	Self-initiated, future-oriented action aimed at improving work situations or changing the status quo	Discretionary extra-role behavior that supports the social and functional environment of the organization	Acquisition, development, and application of knowledge and skills for improvement, adaptation, or task mastery
Relation to organizational standards	Central; explicitly tied to formal evaluation standards, promotion criteria, and organizationally valued pathways of excellence	May support organizational goals, but is not necessarily defined by formal standards or advancement criteria	Generally supportive of organizational functioning, but not necessarily anchored in formal evaluation or promotion systems	May be job- or organization-relevant, but is not inherently defined by formal standards of excellence
Learning / capability development	Core component of the construct	May facilitate initiative, but is not a defining component	Secondary or incidental rather than constitutive	Defining component of the construct
Public enactment / display	Explicitly emphasizes visible enactment, recognition, sharing, contribution, and demonstration of excellence in practice	Emphasizes initiative and change-oriented action, but public recognition is not a necessary defining feature	Often visible in interpersonal or helping behavior, but public display is not its defining logic	May remain internal or developmental; visible display is not required

Dimension	EDB	Proactive Behavior	OCB	Learning Behavior
Typical outcome focus	Recognized performance, promotion, high-quality contribution, sharing, and institutionally valued excellence	Improvement, change, initiative, anticipatory adaptation, or problem prevention	Cooperation, contextual performance, and smoother organizational functioning	Knowledge gain, skill improvement, adaptation, and competence development
Why it differs from excellence-driven behavior	—	Less explicitly tied to institution-specific standards of excellence and formal advancement systems	Not centered on standards clarification and capability-to-excellence conversion	Treats learning as a core endpoint, whereas excellence-driven behavior treats learning as part of a broader enactment process
Representative references	Zhao et al. (2012a); Liu et al. (2013); Hou et al. (2018)	Baer & Frese (2003); Griffin et al. (2007)	Organ (1988); Podsakoff et al. (2000)	Peng et al. (2022)

References

Baer, M., & Frese, M. (2003). Innovation is not enough: Climates for initiative and psychological safety, process innovations, and firm performance. Journal of Organizational Behavior, 24(1), 45–68. https://doi.org/10.1002/job.179

Griffin, M. A., Neal, A., & Parker, S. K. (2007). A new model of work role performance: Positive behavior in uncertain and interdependent contexts. Academy of Management Journal, 50(2), 327–347. https://doi.org/10.5465/AMJ.2007.24634438

Organ, D. W. (1988). Organizational citizenship behavior: The good soldier syndrome. Lexington Books.

Podsakoff, P. M., MacKenzie, S. B., Paine, J. B., & Bachrach, D. G. (2000). Organizational citizenship behaviors: A critical review of the theoretical and empirical literature and suggestions for future research. Journal of Management, 26(3), 513–563. https://doi.org/10.1177/014920630002600307
